# Spatial Remodeling of the Immune Microenvironment During Laryngeal Carcinogenesis Reveals Prognostic Immune Patterns in Laryngeal Squamous Cell Carcinoma

**DOI:** 10.3390/cancers18193130

**Published:** 2026-09-27

**Authors:** Toni Vidović, Filip Tudor, Blažen Marijić, Maximilian Pfeiler, Ita Hadžisejdić, Emina Babarović

**Affiliations:** 1 Department of Otorhinolaryngology, Head and Neck Surgery, Clinical Hospital Center Rijeka, 51000 Rijeka, Croatia; toni.vidovic@uniri.hr (T.V.); filip.tudor@uniri.hr (F.T.); blazen.marijic@uniri.hr (B.M.); 2 Faculty of Medicine, University of Rijeka, 51000 Rijeka, Croatia; max.pfeiler@gmx.at (M.P.); ita.hadzisejdic@uniri.hr (I.H.); 3 Clinical Department of Pathology and Cytology, Clinical Hospital Center Rijeka, 51000 Rijeka, Croatia

**Keywords:** laryngeal squamous cell carcinoma, laryngeal dysplasia, tumor immune microenvironment, immune-cell spatial distribution, FOXP3, CTLA-4, PD-1, tumor-associated macrophages, Protective Immune Score, prognosis

## Abstract

Background/Objectives: Despite therapeutic advances, laryngeal squamous cell carcinoma (LSCC) remains a clinical challenge. We investigated immune microenvironment remodeling and its prognostic relevance during laryngeal carcinogenesis. Methods: This retrospective study included 227 patients: 45 with benign laryngeal polyps, 55 with laryngeal epithelial dysplasia, and 127 with LSCC; 55 (43.3%) LSCCs were Union for International Cancer Control (UICC) stage I–II and 72 (56.7%) stage III–IV. Immunohistochemistry on tissue microarrays (TMAs) was used to assess the density of cluster of differentiation (CD)4-, CD8-, forkhead box P3 (FOXP3)-, programmed cell death protein 1 (PD-1)-, cytotoxic T-lymphocyte-associated protein 4 (CTLA-4)-, CD68-, and CD163-positive immune cells, together with FOXP3/CTLA-4 double-positive cells. Immune-cell densities were expressed as the percentage of the respective intraepithelial/intratumoral or stromal compartment occupied by positively stained immune cells. An exploratory Protective Immune Score (PIS; 0–2) assigned one point each for high intraepithelial FOXP3 and high stromal CTLA-4 cell density. Results: LSCC showed increased stromal immune-cell density and coordinated immune organization. Stromal PD-1 correlated with CD4, CD8, and FOXP3 density (r_s_ = 0.561, 0.515, and 0.403), and stromal CTLA-4 with FOXP3 (r_s_ = 0.556; all *p* < 0.0001). High intraepithelial FOXP3 was associated with improved disease-specific survival (DSS; hazard ratio [HR] = 0.26, 95% confidence interval [CI] 0.10–0.72) and disease-free survival (DFS; HR = 0.24, 95% CI 0.10–0.59). High stromal CTLA-4 similarly favored DSS (HR = 0.29, 95% CI 0.11–0.74) and DFS (HR = 0.26, 95% CI 0.11–0.61). The PIS remained independently associated with DSS (adjusted HR = 0.29, 95% CI 0.16–0.55) and DFS (adjusted HR = 0.29, 95% CI 0.17–0.52). Conclusions: Laryngeal carcinogenesis is characterized by progressive, compartment-specific immune remodeling, with the stroma emerging as the principal site of coordinated immune organization. The PIS captures spatially defined favorable immune features and warrants validation in independent cohorts.

## Simple Summary

Laryngeal squamous cell carcinoma remains difficult to treat, and new biomarkers are needed to identify patients at higher risk of recurrence and disease-related death. We separately analyzed immune cells within the epithelium or tumor as well as surrounding stroma in benign laryngeal lesions, dysplasia, and invasive cancer. Immune cell changes were already present in dysplasia, and stroma showed the strongest coordinated immune organization during cancer progression. Higher density of intraepithelial FOXP3-positive cells and stromal CTLA-4-positive cells were associated with better outcome, whereas higher intraepithelial CD68-positive macrophage density was associated with poorer disease-specific survival. Based on these findings, we developed a Protective Immune Score combining intraepithelial FOXP3 and stromal CTLA-4-positive cells, which was independently associated with disease-free and disease-specific survival. These results highlight the importance of immune-cell localization and coordination and support further validation of this score in independent patient cohorts.

## 1. Introduction

Laryngeal squamous cell carcinoma (LSCC) accounts for approximately 25% of all head and neck squamous cell carcinomas (HNSCCs) and remains one of the most common malignancies of the upper aerodigestive tract [1]. Although its incidence has declined over recent decades, primarily owing to reduced tobacco consumption, survival has improved only marginally, particularly in patients with advanced disease [2,3,4,5,6,7]. Tobacco smoking and alcohol consumption remain the principal etiological factors, while human papillomavirus infection, *Helicobacter pylori* infection, and gastroesophageal and laryngopharyngeal reflux have also been implicated in laryngeal carcinogenesis [8]. Laryngeal squamous lesions comprise a continuous spectrum ranging from benign lesions and epithelial dysplasia to invasive carcinoma. Although the risk of malignant transformation increases with the severity of dysplasia, the biological behavior of individual lesions remains unpredictable, and conventional histopathological grading alone is insufficient for accurate risk stratification [9,10]. Consequently, there is a growing need for biomarkers that better reflect the molecular and immunological changes accompanying laryngeal carcinogenesis. Tumor progression is accompanied by extensive remodeling of tumor microenvironment (TME), which consists of malignant cells and diverse stromal and immune-cell populations that collectively regulate tumor growth, immune escape, and therapeutic response [11]. Among these, cluster of differentiation 4+ (CD4+) helper T lymphocytes, CD8+ cytotoxic T lymphocytes, forkhead box P3+ (FOXP3+) regulatory T cells, and tumor-associated macrophages are key regulators of antitumor immunity and immune suppression. CD68 identifies the overall macrophage population, whereas CD163 predominantly labels M2-like macrophages, which have been associated with tumor progression and unfavorable clinical outcomes in HNSCC [12,13]. However, the interactions among these immune-cell populations during the progression from benign lesions through dysplasia to invasive LSCC remain poorly understood. The adaptive immune response is further regulated by immune checkpoint pathways, among which programmed cell death protein-1 (PD-1) and cytotoxic T-lymphocyte-associated antigen-4 (CTLA-4) are the best characterized [14,15]. These inhibitory receptors suppress T-cell activation and promote peripheral immune tolerance, thereby limiting excessive immune responses under physiological conditions. Within the tumor microenvironment, persistent activation of PD-1 and CTLA-4 contributes to functional exhaustion of effector T lymphocytes and facilitates tumor immune escape. Although CTLA-4 primarily regulates early T-cell activation, whereas PD-1 predominantly modulates immune responses in peripheral tissues, both pathways represent central mechanisms of cancer-associated immunosuppression [14,15,16]. FOXP3+ regulatory T cells constitute a specialized subset of CD4+ lymphocytes with potent immunosuppressive properties. These cells suppress the activity of effector T lymphocytes through multiple mechanisms, including high constitutive expression of CTLA-4, thereby promoting immune tolerance within the tumor microenvironment [17]. Consequently, the balance between FOXP3+ Tregs and CD8+ cytotoxic T lymphocytes has emerged as important determinant of antitumor immunity. However, despite extensive investigation, the prognostic significance of Treg infiltration remains controversial. Previous studies have reported conflicting findings, with some demonstrating associations between increased Treg infiltration and poor prognosis, whereas others observed improved disease-free or overall survival in patients with HNSCC [18,19,20,21,22,23,24,25]. These discrepancies likely reflect differences in tumor site, molecular characteristics, disease stage, and, importantly, the spatial distribution of immune-cell populations within the tumor microenvironment. The introduction of immune checkpoint inhibitors targeting the PD-1 pathway has substantially changed the treatment landscape of recurrent and metastatic HNSCC, resulting in improved survival and reduced treatment-related toxicity in selected patients [26]. Nevertheless, durable clinical responses are achieved in only a subset of patients, indicating that currently available biomarkers remain insufficient for accurate patient selection. A better understanding of the immune microenvironment and its remodeling during tumor progression is therefore essential for the identification of novel prognostic and predictive biomarkers, as well as potential therapeutic targets [27,28,29,30]. Despite growing interest in cancer immunology, the immune alterations accompanying the progression from benign laryngeal lesions through epithelial dysplasia to invasive LSCC remain insufficiently characterized. In particular, studies simultaneously evaluating immune checkpoint molecules, regulatory T cells, macrophage populations, helper and cytotoxic T lymphocytes, and their spatial distribution within epithelial and stromal compartments throughout the entire process of laryngeal carcinogenesis are scarce. Accordingly, the aim of this study was to comprehensively characterize immune microenvironment remodeling during laryngeal carcinogenesis by evaluating the density of PD1-positive cells, CTLA4-positive cells, FOXP3-positive cells, FOXP3/CTLA4 double-positive cells, CD4-positive cells, CD8-positive cells, CD68-positive macrophages, and CD163-positive macrophages in benign laryngeal polyps, premalignant squamous lesions, and invasive LSCC. Furthermore, we investigated their spatial distribution within epithelial and stromal compartments, evaluated correlations among these immune-cell populations, and assessed their associations with clinicopathological characteristics and patient outcomes.

## 2. Materials and Methods

### 2.1. Study Cohort

This retrospective study was conducted using archived formalin-fixed paraffin-embedded (FFPE) tissue samples obtained from the Clinical Department of Pathology and Cytology, Clinical Hospital Center Rijeka, Rijeka, Croatia. The study cohort comprised patients treated for laryngeal lesions at the Department of Otorhinolaryngology and Head and Neck Surgery, Clinical Hospital Center Rijeka, Rijeka, Croatia, between 2010 and 2022. A total of 227 laryngeal tissue samples were included: 127 from patients with laryngeal squamous cell carcinoma (LSCC), 55 from patients with premalignant squamous epithelial lesions (low- and high-grade dysplasia), and 45 from patients with benign laryngeal polyps, which served as the control group. Patients were included in the study depending on whether complete clinicopathological data and sufficient preserved tissue were available for immunohistochemical analysis. Clinical characteristics of patients with benign laryngeal polyps and laryngeal epithelial dysplasia are provided in Supplementary Table S1. Eligible patients had histologically confirmed low- or high-grade laryngeal squamous dysplasia or primary invasive LSCC and had not received previous oncological treatment, including radiotherapy or chemotherapy. Patients with insufficient preserved tissue, previous oncological treatment, malignancies originating from the oropharynx or hypopharynx, or synchronous primary malignancies were excluded. Clinicopathological data, including age at diagnosis, sex, smoking status, alcohol consumption, tumor grade, lymphovascular invasion, perineural invasion, tumor–node–metastasis (TNM) stage, recurrence, and survival, were retrieved from the pathology database and the hospital information system. Tumors were staged according to the 8th edition of the AJCC/UICC TNM classification [31]. For stage-stratified analyses, patients were categorized as having early-stage (UICC stage I–II) or advanced-stage (UICC stage III–IV) disease. Pathological T classification (pT1–2 vs. pT3–4), pathological nodal status (pN0 vs. pN+), and UICC stage (I–II vs. III–IV) were additionally evaluated as separate clinicopathological variables in the Cox regression analyses.

Sample size estimation was performed using G*Power software (version 3.1.9.7; Heinrich-Heine University Düsseldorf, Düsseldorf, Germany) based on preliminary PD-1 immunohistochemical data obtained from patients with invasive LSCC and premalignant laryngeal lesions. Assuming a type I error of 0.05 and a statistical power of 80% (type II error = 0.20), the calculated sample size confirmed the adequacy of the study cohort. The study was approved by the Ethical Committee of the Clinical Hospital Center Rijeka, Rijeka, Croatia. All procedures were performed in accordance with the principles of the Declaration of Helsinki, Good Clinical Practice guidelines, and applicable national regulations regarding research involving human biological material and patient confidentiality.

### 2.2. Tissue Microarray Construction

New histological sections were prepared from the previously selected paraffin-embedded tissue blocks containing the lesion or tumor and stained with hematoxylin and eosin (H&E). A pathologist reviewed the slides, confirmed the diagnosis, and identified representative areas of the lesion or tumor suitable for inclusion in the study. The selected areas were marked on the histological slides with a waterproof marker, after which the corresponding regions were identified and marked on the respective donor paraffin blocks. Tissue microarray (TMA) blocks were constructed using MTA Booster OI manual tissue microarrayer (Alphelys, Plaisir, France). Three 1 mm cores of representative tissue were obtained from each donor paraffin block and transferred into a new recipient paraffin block. The cores were arranged according to a predefined coordinate system along the x- and y-axes, with an inter-core spacing of 0.5 mm. Normal liver or placental tissue was used as an orientation core within each TMA block. Following construction, the recipient TMA blocks were incubated overnight at 45 °C to promote adhesion and incorporation of the tissue cores into the surrounding paraffin. The prepared blocks were subsequently sectioned for further histological and immunohistochemical analyses. Representative immunohistochemical staining of benign mucosa, dysplasia and invasive LSCC are shown in Figure 1.

### 2.3. Immunohistochemical Analysis

Three to four μm thick sections were cut from the paraffin-embedded TMA blocks and mounted on silanized adhesive microscope slides (VitroGnost SIL, BioGnost, Zagreb, Croatia) to enhance tissue adhesion. The slides were dried overnight at 37 °C and subsequently stored at 4 °C until immunohistochemical staining. Before immunohistochemical analysis, the TMA sections were deparaffinized in issue-Tek Tissue-Clear (Sakura Finetek Europe B.V., Alphen aan den Rijn, The Netherlands) and rehydrated through graded ethanol concentrations according to the standard laboratory protocol. Following deparaffinization, heat-induced epitope retrieval (HIER) was performed in a PT Link system (Dako, Agilent Technologies, Glostrup, Denmark) at 97 °C for 20 min. For PD-1 immunohistochemical staining, an additional Rabbit Linker step (Dako, Agilent Technologies, Glostrup, Denmark) was applied after HIER to increase the sensitivity of the reaction, in accordance with the manufacturer’s recommendations and the validated laboratory protocol. Immunohistochemical staining for CTLA-4, PD-1, FOXP3, CD4, CD8, and CD68 was performed using a Dako Autostainer Plus automated platform (DakoCytomation, Fort Collins, CO, USA), whereas CD163 staining was performed using a Ventana BenchMark ULTRA platform (Ventana Medical Systems, Roche Diagnostics, Tucson, AZ, USA). All staining procedures were performed according to previously validated laboratory protocols. Following the blocking of endogenous peroxidase activity, the slides were incubated with primary antibodies at the appropriate dilutions and for the predetermined incubation times. The corresponding detection system was then applied, with diaminobenzidine used as the chromogen. Finally, the slides were counterstained with hematoxylin, dehydrated through increasing concentrations of ethanol, cleared in xylene, and cover-slipped. The following primary antibodies and epitope retrieval were used: CTLA-4 (clone sc-376016, Santa Cruz Biotechnology, Dallas, TX, USA; dilution 1:100; PT Link; 30 min incubation), PD-1 (clone EPR4877(2), Abcam, Cambridge, UK; dilution 1:500; PT Link and Rabbit Linker; 30 min incubation), FOXP3 (clone 236A/E7, Abcam, Cambridge, UK; dilution 1:200; PT Link; 60 min incubation), CD4 (clone SP35, Cell Marque, Rocklin, CA, USA; dilution 1:25; PT Link; 30 min incubation), CD8 (clone C8/144B, Dako Agilent, Santa Clara, CA, USA; ready-to-use antibody; PT Link; 30 min incubation), CD68 (clone PG-M1, Dako Agilent, Santa Clara, CA, USA; dilution 1:100; PT Link; 30 min incubation). CD163 immunohistochemistry was performed separately on the Ventana BenchMark ULTRA platform using the CD163 antibody (clone 10D6, Leica Biosystems, Buffalo Grove, IL, USA; dilution 1:25) according to the laboratory-validated Ventana staining protocol. To identify cells simultaneously positive for FOXP3 and CTLA-4, double immunohistochemical staining was performed using the DAKO EnVision™ Doublestain System (K5361; Dako, Glostrup, Denmark). Nuclear FOXP3 positivity was visualized using a brown DAB chromogen, whereas cytoplasmic CTLA-4 positivity was visualized using a red chromogen. This approach enabled the identification of FOXP3/CTLA-4 double-positive cells with a regulatory T-lymphocyte phenotype. Appropriate positive and negative controls were included in all immunohistochemical staining procedures. In the negative controls, the primary antibody was replaced with the corresponding buffer solution. Following immunohistochemical staining, all TMA slides were digitized using a PreciPoint Fritz Histo Scanner (PreciPoint GmbH, Garching, Germany) equipped with a Nikon CFI Plan Apochromat 20× objective (Nikon Instruments Inc., Tokyo, Japan) to enable standardized digital pathological evaluation.

### 2.4. Evaluation of Immunohistochemical Staining

Immunohistochemical evaluation was performed according to the recommendations of the International Immuno-Oncology Biomarker Working Group for the assessment of tumor-infiltrating lymphocytes (TILs) in solid tumors [32], with minor adaptations for TMA-based analysis. All immunohistochemically stained TMA slides were independently evaluated by two pathologists blinded to the clinicopathological characteristics and patient outcomes. For each biomarker tested, immune cell infiltration was quantified separately within the intraepithelial/intratumoral compartment (immune cells located within epithelial dysplastic lesions or tumor cell nests) and the stromal compartment (immune cells located in the peritumoral stroma within approximately 1 mm of the epithelial/tumor component). Areas of necrosis, tissue artifacts, and non-representative cores were excluded from analysis. Each case was represented by three independent 1 mm TMA cores. For every core, the percentage of tissue area occupied by immunohistochemically positive immune cells was estimated separately for the intraepithelial/intratumoral and stromal compartments. The final value for each biomarker and compartment for each case was calculated as the mean percentage across the three evaluable cores. Accordingly, immune cell density values reported throughout the study are expressed as percentages (%) of the respective tissue compartment occupied by positively stained immune cells.

### 2.5. Statistical Analysis

Statistical analyses were performed using MedCalc Statistical Software version 23.6.1 (MedCalc Software Ltd., Ostend, Belgium). Continuous variables were expressed as medians with ranges or interquartile ranges (IQRs), and categorical variables as frequencies and percentages. Since the majority of continuous variables deviated from a normal distribution (D’Agostino–Pearson test, *p* < 0.05), and because several subgroup analyses involved relatively small sample sizes, non-parametric statistical methods were used throughout the study, as they were considered more appropriate than their parametric counterparts.

Comparisons among the three study groups (benign lesions, premalignant lesions, and LSCC) were performed using the Kruskal–Wallis test, and, when appropriate, pairwise comparisons were performed using Conover’s post hoc test.

Comparisons between two independent groups were performed using the Mann–Whitney U test. Associations between categorical variables were evaluated using the χ^2^ test or Fisher’s exact test. Correlations between immune biomarkers were evaluated using Spearman’s rank correlation coefficient because immune-cell density variables were predominantly non-normally distributed and Spearman’s method does not assume normally distributed variables or a linear relationship between variables. Correlation matrices were additionally visualized as heatmaps.

Disease-specific survival (DSS) was defined as the interval between the date of diagnosis and disease-specific death, with patients alive at the last follow-up and those who died from other causes censored at the date of last follow-up or death, respectively. Disease-free survival (DFS) was defined as the interval between the date of diagnosis and the first documented disease recurrence or the last follow-up in patients without recurrence. The median follow-up duration was estimated using the reverse Kaplan–Meier method, with patients alive at the last follow-up treated as events and deaths from any cause censored.

For survival analyses, patients were dichotomized into low- and high-density groups for each immune biomarker using the respective median value as the cut-off. Survival probabilities were estimated using the Kaplan–Meier method and compared using the log-rank test. Numbers at risk were displayed below all Kaplan–Meier survival curves. Kaplan–Meier curves were graphically truncated at 120 months because of the small number of patients remaining at risk beyond this time point; however, all available follow-up data were retained in the survival analyses. The prognostic significance of clinicopathological and immunological variables was further evaluated using univariate and multivariate Cox proportional hazards regression models, and the results were reported as hazard ratios (HRs) with 95% confidence intervals (CIs). All statistical tests were two-sided, and a *p*-value < 0.05 was considered statistically significant.

## 3. Results

### 3.1. Study Cohort

The study cohort comprised 227 patients, including 45 patients with benign laryngeal polyps serving as controls, 55 patients with laryngeal epithelial dysplasia, and 127 patients with LSCC. Clinical characteristics of the control and dysplasia groups are summarized in Supplementary Table S1, whereas the detailed clinicopathological characteristics of patients with LSCC are presented in Table 1. Among patients with laryngeal epithelial dysplasia, 29 (52.7%) had low-grade and 26 (47.3%) had high-grade dysplasia. During follow-up, 6 patients (10.9%) progressed to invasive LSCC, whereas 49 (89.1%) showed no progression.

Among patients with LSCC, most were male (92.1%) and older than 60 years (64.6%). The median age at diagnosis was 63.2 years, ranging from 43.9 to 87.4 years. Approximately half of the patients reported alcohol consumption (49.6%), and the majority were smokers (78.0%). Patients were relatively evenly distributed across pT categories, although pT3 tumors were the most frequent (37.8%). For stage-stratified analyses, UICC stages I–II were classified as early-stage and UICC stages III–IV as advanced-stage disease. In the present cohort, dichotomized pT classification (pT1–2 vs. pT3–4) showed near-complete agreement with the early-stage (UICC stage I–II) versus advanced-stage (UICC stage III–IV) classification (125/127 cases, 98.4%; Cohen’s κ = 0.968, *p* < 0.0001).

Most patients had no regional lymph node metastases (pN0, 78.7%) or distant metastases (M0, 97.6%). Overall, 72 patients (56.7%) had advanced-stage disease, whereas 55 (43.3%) had early-stage LSCC. Histologically, moderately differentiated tumors (G2) predominated (48.0%), while lymphovascular and perineural invasion were identified in 35.4% and 11.8% of evaluable cases, respectively. Neck dissection was performed in 29.1% of patients, and postoperative radiotherapy (PORT) was administered to 52.8%, whereas 46.5% received surgery alone. During follow-up, disease recurrence occurred in 29 patients (22.8%), with local recurrence being the most frequent pattern (11.8%). At the last follow-up, 69 patients (54.3%) were alive, 23 (18.1%) had died of LSCC, and 35 (27.6%) had died of other causes. The median follow-up duration, estimated using the reverse Kaplan–Meier method, was 113.3 months (95% CI, 91.5–128.0 months). A second primary malignancy was diagnosed in 31 patients (24.4%), predominantly outside the head and neck region (74.2%).

### 3.2. Progressive Remodeling of the Immune Microenvironment Across Normal Mucosa, Dysplasia and Invasive SCC

A progressive remodeling of the immune microenvironment was observed across the spectrum of laryngeal carcinogenesis (Table 2). Adaptive immune cell infiltration differed significantly among the study groups. Within the intraepithelial compartment, CD4 and CD8-positive cell density was significantly higher in dysplastic lesions and invasive SCC than in normal mucosa (both *p* < 0.01), whereas no significant differences were observed between dysplasia and SCC. In contrast, stromal CD4- and CD8-positive cell density were markedly increased in invasive SCC, with significantly higher density than in both, normal mucosa and dysplastic lesions (both *p* < 0.0001), while no differences were detected between the control and dysplasia groups.

Macrophage infiltration showed a similar progressive pattern during laryngeal carcinogenesis. Intraepithelial CD68-positive macrophage density showed a stepwise increase from normal mucosa to dysplasia and SCC (*p* < 0.0001), whereas intraepithelial CD163-positive macrophage density was significantly higher only in SCC than in the other two groups. Stromal and overall CD68- and CD163-positive macrophage density was markedly increased in SCC compared with both control and dysplastic tissue (all *p* < 0.0001).

Immune checkpoint and regulatory markers also demonstrated progressive changes during laryngeal carcinogenesis. PD1-positive cell density increased progressively across laryngeal carcinogenesis, particularly within the stromal and overall compartments, where a stepwise increase from control through dysplasia to SCC was observed (both *p* < 0.0001). Intraepithelial CTLA4-positive cell density was significantly higher in dysplasia and SCC than in controls, whereas stromal and overall CTLA4-positive cell density was significantly increased only in SCC. FOXP3-positive cell density did not differ between groups in the intraepithelial compartment but was markedly increased in the stromal and overall compartments of SCC (both *p* < 0.0001).

### 3.3. Immune Cell Density According to Dysplasia Grade and Progression to SCC

Immune cell density differed according to the grade of dysplasia (Table 3). High-grade dysplasia exhibited significantly higher stromal and overall CD4-positive cell density than low-grade dysplasia (*p* = 0.012 and *p* = 0.016, respectively), whereas CD8-positive cell density did not differ between the two groups. Among macrophage markers, overall CD68-positive macrophage density was significantly increased in high-grade lesions (*p* = 0.011), while a trend toward increased intraepithelial CD68-positive macrophage density was observed (*p* = 0.051). The most pronounced differences were observed for immune checkpoint and regulatory markers. High-grade dysplasia demonstrated significantly higher overall FOXP3-positive cell density (*p* = 0.041) and a trend toward increased stromal FOXP3-positive cell density (*p* = 0.052), accompanied by significantly higher stromal and overall FOXP3/CTLA4 double-positive cell density (*p* = 0.039 and *p* = 0.045, respectively). PD1-positive cell density was consistently higher in high-grade dysplasia across the intraepithelial, stromal, and overall compartments (*p* = 0.005, *p* = 0.004, and *p* = 0.001, respectively).

### 3.4. Immune Cell Composition Between Early- and Advanced-Stage SCC

The immune cell composition differed between early- and advanced-stage SCC (Table 4). Advanced-stage SCC exhibited significantly higher stromal CD8-positive cell density than early-stage tumors (*p* = 0.010), whereas stromal CD4-positive cell density did not differ between the two groups. Among macrophage markers, advanced-stage SCC demonstrated significantly higher intraepithelial CD68-positive macrophage density (*p* = 0.028) and CD163-positive macrophage density (*p* = 0.032), while stromal CD68- and CD163-positive macrophage density remained comparable between early- and advanced-stage tumors. Regarding immune checkpoint and regulatory markers, stromal FOXP3-positive cell density was significantly higher in advanced-stage SCC (*p* = 0.006). Stromal FOXP3/CTLA4 double-positive cell density (*p* = 0.104) and stromal PD1-positive cell density (*p* = 0.199) also tended to be higher in advanced-stage tumors, although these differences did not reach statistical significance. No significant differences were observed in intraepithelial FOXP3-positive, FOXP3/CTLA4 double-positive, or PD1-positive cell density between the two groups.

### 3.5. Compartment-Specific Immune Correlation Networks During Laryngeal Carcinogenesis

A progressive, compartment-specific organization of the immune microenvironment was observed across the spectrum of laryngeal carcinogenesis (Figure 2). The stromal compartment demonstrated substantially stronger and more extensive positive correlations among immune markers than the intraepithelial/intratumoral compartment, indicating a more coordinated immune network. Across all samples, stromal PD1-positive cell density showed strong positive correlations with CD4- and CD8-positive cell density, while FOXP3-positive cell density closely correlated with CTLA4 and FOXP3/CTLA4 double-positive cell density. In contrast, the intraepithelial/intratumoral compartment exhibited fewer and generally weaker associations, resulting in a more fragmented correlation pattern. Coordinated stromal immune correlation networks were already evident in dysplastic lesions, particularly between PD1 and CD4, PD1 and CD8, and between FOXP3/CTLA4 double-positive cell density and macrophage markers. In invasive SCC, these stromal associations became more extensive and pronounced, with strengthened correlations involving PD1, FOXP3, CD4, CD8, and immune checkpoint markers, whereas the intraepithelial/intratumoral compartment remained comparatively less interconnected.

Consistent with the correlation heatmaps, stromal correlations were generally stronger than those observed in the intraepithelial compartment. The strongest stromal and intraepithelial immune correlations are summarized in Table 5. Across all samples, stromal PD1-positive cell density demonstrated strong positive correlations with CD4 (r_s_ = 0.561, *p* < 0.0001), CD8 (r_s_ = 0.515, *p* < 0.0001), and FOXP3 (r_s_ = 0.403, *p* < 0.0001), while CTLA4 positively correlated with FOXP3 (r_s_ = 0.556, *p* < 0.0001). Similar stromal correlation networks were already established in dysplastic lesions, with particularly strong correlations between PD1 and CD4 (r_s_ = 0.654, *p* = 0.0001), PD1 and CD8 (r_s_ = 0.521, *p* = 0.003), and between FOXP3/CTLA4 double-positive cell density and CD68-positive macrophage density (r_s_ = 0.530, *p* = 0.0009). The strongest correlation identified in the study was observed in high-grade dysplasia between stromal FOXP3/CTLA4 double-positive cell density and CD68-positive macrophage density (r_s_ = 0.752, *p* = 0.001). In LSCC, strong stromal correlations between CTLA4 and FOXP3 persisted in both early- and advanced-stage tumors, while advanced-stage SCC additionally demonstrated a significant association between stromal PD1-positive and CD4-positive cell density (r_s_ = 0.456, *p* = 0.0002). Complete results of the compartment-specific immune correlation analyses are provided in Supplementary Table S2.

### 3.6. Survival Analysis

#### 3.6.1. Disease-Specific Survival

Kaplan–Meier survival analysis demonstrated that, in the overall LSCC cohort, high intratumoral CD68-positive macrophage density was associated with significantly shorter DSS than low intratumoral CD68-positive macrophage density (*p* = 0.015, Figure 3A). In contrast, high intratumoral FOXP3-positive cell density and high stromal CTLA4-positive cell density were associated with significantly prolonged DSS (*p* = 0.005 and *p* = 0.006, respectively; Figure 3B,C). Among patients with advanced-stage LSCC, high intratumoral FOXP3-positive cell density and high stromal CTLA4-positive cell density remained significantly associated with prolonged DSS (*p* = 0.003 and *p* = 0.0006, respectively; Figure 3E,F), while high intratumoral CD68-positive macrophage density showed a trend toward shorter DSS (*p* = 0.070, Figure 3D). Several additional biomarkers demonstrated a trend toward longer DSS, including stromal FOXP3-positive cell density (*p* = 0.077), intratumoral FOXP3/CTLA4 double-positive cell density (*p* = 0.068), and stromal FOXP3/CTLA4 double-positive cell density (*p* = 0.076). Patients with early-stage LSCC were not analyzed separately for DSS because only one disease-specific death occurred in this subgroup, precluding a meaningful survival analysis. Complete results of the Kaplan–Meier DSS analyses are provided in Supplementary Table S3, whereas Kaplan–Meier curves for the principal associations in the overall and advanced-stage LSCC cohorts are shown in Figure 3.

Univariate Cox proportional hazards regression analysis identified both pT3–4 and UICC stage III–IV as significant adverse prognostic factors for DSS (for both, HR = 19.71, 95% CI 2.65–146.30, *p* = 0.004). Positive lymph node status, pN+ status (HR = 3.30, 95% CI 1.44–7.52, *p* = 0.005), lymphovascular invasion (HR = 3.34, 95% CI 1.26–8.80, *p* = 0.015), and receipt of postoperative radiotherapy (HR = 3.39, 95% CI 1.26–9.13, *p* = 0.016) were also associated with poorer DSS. Histological grade G2–G3 (HR = 3.87, 95% CI 0.90–16.63, *p* = 0.069) and perineural invasion (HR = 2.46, 95% CI 0.89–6.85, *p* = 0.084) showed a statistical trend. Among the investigated immune biomarkers, high intratumoral CD68-positive macrophage density was associated with an increased risk of disease-specific death (HR = 3.03, 95% CI 1.18–7.77, *p* = 0.021), whereas high intratumoral FOXP3-positive cell density (HR = 0.26, 95% CI 0.10–0.72, *p* = 0.009) and high stromal CTLA4-positive cell density (HR = 0.29, 95% CI 0.11–0.74, *p* = 0.010) were associated with significantly improved DSS. No significant associations were observed for CD163-positive macrophage density, PD1-positive cell density, FOXP3/CTLA4 double-positive cell density, or stromal FOXP3-positive cell density (Table 6).

#### 3.6.2. Disease-Free Survival

In the overall LSCC cohort, high intratumoral FOXP3-positive cell density was associated with significantly prolonged DFS compared with low intratumoral FOXP3-positive cell density (*p* = 0.0008, Figure 4A). Similarly, patients with high stromal CTLA4-positive cell density demonstrated significantly longer DFS (*p* = 0.0009, Figure 4B), while high intratumoral FOXP3/CTLA4 double-positive cell density was also associated with improved DFS (*p* = 0.020, Figure 4C). In addition, stromal FOXP3-positive cell density (*p* = 0.064) and intratumoral CTLA4-positive cell density (*p* = 0.089) showed a trend toward longer DFS.

Among patients with advanced-stage LSCC, high intratumoral FOXP3-positive cell density (*p* = 0.0008, Figure 4D), high stromal CTLA4-positive cell density (*p* = 0.0003, Figure 4E), and high intratumoral FOXP3/CTLA4 double-positive cell density (*p* = 0.026, Figure 4F) remained significantly associated with prolonged DFS. Stromal FOXP3-positive cell density (*p* = 0.061) and stromal FOXP3/CTLA4 double-positive cell density (*p* = 0.091) showed non-significant associations in the same direction. No statistically significant associations were observed in patients with early-stage LSCC; however, only seven patients developed disease recurrence in this subgroup, substantially limiting statistical power. No significant associations with DFS were observed for CD163-positive macrophage density or PD1-positive cell density in either the overall LSCC cohort or stage-specific subgroup analyses. Complete results of the Kaplan–Meier DFS analyses are provided in Supplementary Table S4, whereas Kaplan–Meier curves for the principal statistically significant associations in the overall and advanced-stage LSCC cohorts are shown in Figure 4.

Univariate Cox proportional hazards regression analysis identified UICC stage III–IV as a significant predictor of disease recurrence (HR = 2.30, 95% CI 1.02–5.20, *p* = 0.045). pN+ status (HR = 1.98, 95% CI 0.90–4.36, *p* = 0.088) and lymphovascular invasion (HR = 2.17, 95% CI 0.93–5.08, *p* = 0.075) were associated with an increased hazard of recurrence but did not reach statistical significance. Among the investigated immune biomarkers, high intratumoral FOXP3-positive cell density was significantly associated with a reduced risk of recurrence (HR = 0.24, 95% CI 0.10–0.59, *p* = 0.002). Likewise, high stromal CTLA4-positive cell density was associated with a lower risk of recurrence (HR = 0.26, 95% CI 0.11–0.61, *p* = 0.002), while high intratumoral FOXP3/CTLA4 double-positive cell density was also significantly associated with a reduced recurrence risk (HR = 0.27, 95% CI 0.08–0.88, *p* = 0.030). Stromal FOXP3-positive cell density (HR = 0.48, 95% CI 0.21–1.06, *p* = 0.070) and intratumoral CTLA4-positive cell density (HR = 0.46, 95% CI 0.19–1.15, *p* = 0.098) demonstrated borderline significance. No significant associations were observed for CD68-positive macrophage density, CD163-positive macrophage density, or PD1-positive cell density (Table 7).

#### 3.6.3. Development of the Protective Immune Score

Based on these findings, an exploratory composite Protective Immune Score (PIS) was developed. The score incorporated the two immune biomarkers that consistently demonstrated a favorable prognostic impact in both disease-specific and disease-free survival analyses, namely intratumoral FOXP3-positive cell density and stromal CTLA4-positive cell density. One point was assigned for high intratumoral FOXP3-positive cell density and one point for high stromal CTLA4-positive cell density, resulting in a cumulative score ranging from 0 to 2. The PIS was calculated only for patients with evaluable data for both biomarkers. Among all evaluable cases, 67 patients (38.1%) had a PIS of 0, 65 (36.9%) had a PIS of 1, and 44 (25.0%) had a PIS of 2. Within the LSCC cohort, the corresponding distribution was 39 (34.2%), 39 (34.2%), and 36 (31.6%), respectively (Table 8).

Increasing PIS was associated with progressively improved disease-specific and disease-free survival in both the overall LSCC cohort and patients with advanced-stage disease (Figure 5). Patients with a PIS of 2 consistently demonstrated the most favorable outcomes, whereas those with a PIS of 0 had the poorest survival, supporting the concept of a coordinated protective immune phenotype in LSCC.

#### 3.6.4. Multivariate Analysis for Disease-Specific Survival

To determine whether the identified immune biomarkers retained their prognostic significance after adjustment for established clinicopathological factors, multivariate Cox regression analyses were performed. For disease-specific survival, UICC stage III–IV remained an independent predictor of disease-specific mortality (HR = 18.53, 95% CI 2.38–144.18, *p* = 0.005). The PIS remained independently associated with a significantly reduced risk of disease-specific death (HR = 0.29, 95% CI 0.16–0.55, *p* < 0.001). In contrast, lymph node status was not independently associated with DSS after adjustment for disease stage and the Protective Immune Score (Table 9).

#### 3.6.5. Multivariate Analysis for Disease-Free Survival

Multivariate Cox regression analysis for DFS demonstrated similar findings. The Protective Immune Score remained independently associated with a significantly reduced risk of disease recurrence (HR = 0.29, 95% CI 0.17–0.52, *p* < 0.001). UICC stage III–IV demonstrated a strong trend toward an increased risk of recurrence (HR = 2.57, 95% CI 0.99–6.69, *p* = 0.052), whereas pN+ status was not independently associated with DFS (HR = 1.70, 95% CI 0.70–4.13, *p* = 0.238). The results are summarized in Table 10.

## 4. Discussion

The present study provides a comprehensive evaluation of immune microenvironment remodeling across the spectrum of laryngeal carcinogenesis by integrating compartment-specific immunohistochemical profiling, correlation-network analysis, and survival evaluation in benign laryngeal lesions, dysplasia, and invasive laryngeal squamous cell carcinoma (LSCC). Rather than reflecting a simple shift toward immunosuppression, our findings indicate a progressive spatial reorganization of coordinated immune activation and regulation during laryngeal carcinogenesis. Immune alterations were already detectable in dysplastic lesions, but their temporal patterns differed between tissue compartments: intraepithelial CD4-positive, CD8-positive, PD1-positive, and CTLA4-positive cell density increased early, whereas marked accumulation of T-cell, regulatory, checkpoint-associated, and macrophage populations within the stroma became most evident after invasive transformation. Importantly, the stromal compartment contained substantially stronger and more extensive correlations among PD1-positive, CTLA4-positive, FOXP3-positive, CD4-positive, and CD8-positive cell density and macrophage density than the intraepithelial compartment, identifying the stroma as the principal site of coordinated immune organization. Higher intraepithelial FOXP3-positive cell density and higher stromal CTLA4-positive cell density were associated with longer disease-free survival (DFS) and improved disease-specific survival (DSS), whereas higher intraepithelial CD68-positive macrophage density was associated with poor DSS. The combination of intraepithelial FOXP3-positive and stromal CTLA4-positive cell densities in the Protective Immune Score (PIS) remained independently associated with both DFS and DSS after adjustment for disease stage and lymph node status. Together, these findings indicate that LSCC progression is characterized by the development of an immune-inflamed yet simultaneously immunoregulated microenvironment whose biological and prognostic significance depends on spatial organization, cellular context, and coordinated relationships among immune populations.

One of the central observations was the compartment-specific sequence of immune changes during progression from control lesions through dysplasia to invasive carcinoma. Intraepithelial CD4-positive density and CD8-positive cell density were already higher in dysplastic lesions than in controls, without a further significant increase between dysplasia and SCC. In contrast, stromal and overall CD4-positive and CD8-positive cell density increased predominantly after invasive transformation. This pattern suggests that premalignant epithelial alterations are recognized by the host immune system before invasion, whereas extensive immune-cell recruitment and organization within the stroma accompany malignant transformation. The tumor stroma should therefore not be viewed as a passive scaffold but as a dynamic niche in which immune cells, fibroblasts, endothelial cells, extracellular matrix components, and soluble mediators jointly influence antigen presentation, lymphocyte trafficking, invasion, and immune escape. Ma et al. similarly emphasized the convergence of T-cell dysfunction, checkpoint signaling, macrophage activity, and metabolic reprogramming within the LSCC microenvironment [33]. Our findings extend this concept by showing that immune remodeling evolves in a spatially ordered manner, with early intraepithelial immune recognition followed by increasingly extensive and coordinated stromal organization.

The dysplasia analyses further support the concept that immune remodeling begins before invasive transformation. High-grade dysplasia showed higher stromal and overall CD4-positive cell density, higher overall CD68-positive macrophage density, higher stromal and overall FOXP3-positive and FOXP3/CTLA4 double-positive cell density, and higher PD1-positive cell density in all evaluated compartments than low-grade dysplasia. Dysplasias that subsequently progressed to SCC also showed higher stromal and overall FOXP3-positive cell density and higher intraepithelial FOXP3/CTLA4 double-positive cell density than non-progressing lesions. However, these findings are based on a very small number of dysplasias that progressed to carcinoma and should therefore be interpreted with considerable caution; this limited number of progression events precludes any reliable conclusions regarding predictive validity. Nevertheless, the internal consistency of the observed patterns suggests that increasing histological severity may be accompanied by both stronger immune activation and a stronger regulatory counterbalance. The premalignant laryngeal microenvironment may therefore be neither immunologically quiescent nor uniformly suppressed but actively engaged in immunoediting before invasion.

The correlation analysis presented in Figure 2 adds an important dimension to the quantitative findings and represents one of the distinctive contributions of the present study. Across the full series, stromal PD1-positive cell density correlated strongly with CD4-positive, CD8-positive, and FOXP3-positive cell density, while stromal CTLA4-positive cell density correlated strongly with FOXP3-positive cell density. Coordinated networks were already evident in dysplasia, including correlations between stromal PD1-positive cell density and both CD4-positive and CD8-positive cell density, as well as associations between CTLA4-related cell density and macrophage density. The strongest individual association was observed in high-grade dysplasia between stromal FOXP3/CTLA4 double-positive cell density and CD68-positive macrophage density. Although these correlations do not demonstrate direct cellular interactions, they show that effector, regulatory, checkpoint-associated, and macrophage populations do not accumulate independently. Instead, the findings indicate parallel and coordinated remodeling of adaptive and myeloid immune components. The substantially stronger and more extensive stromal correlation pattern supports the conclusion that the stroma is the principal compartment in which these populations are recruited, organized, and potentially functionally integrated. In contrast, the weaker and more fragmented intraepithelial correlation pattern suggests more selective recruitment within epithelial and tumor nests.

This network-based perspective distinguishes the present study from conventional single-marker immunohistochemical analyses. The biological meaning of an immune population appears to depend not only on its density and compartmental localization but also on the accompanying immune-cell configuration. The strong relationships among CD4-positive, CD8-positive, FOXP3-positive, PD1-positive, and CTLA4-positive cell density indicate the development of a coordinated immune-inflamed state, while the associations with CD68-positive and CD163-positive macrophage density suggest that lymphoid and myeloid remodeling occur in parallel. Figure 2 therefore supports a model of laryngeal carcinogenesis as a process of spatially structured immune reorganization rather than a series of independent changes in individual biomarkers. The complete correlation matrices, including weaker and non-significant associations, are provided in the Supplementary Table S2.

PD1 further illustrates why isolated marker interpretation may be insufficient. Stromal and overall PD1-positive cell density increased progressively from control lesions through dysplasia to SCC, were higher in high-grade than in low-grade dysplasia, and correlated closely with CD4-positive and CD8-positive cell density, particularly in the stroma. Nevertheless, PD1-positive cell density alone was not associated with DFS or DSS. This finding is compatible with the context-dependent nature of PD1, which may identify both activated antigen-experienced T cells and functionally impaired populations. Oliva et al. similarly emphasized that the relevance of PD1-positive T cells in HNSCC depends on the accompanying cytotoxic infiltrate and additional exhaustion markers [34]. Because single-marker immunohistochemistry cannot distinguish these functional states, the absence of a survival association does not indicate biological irrelevance but suggests that PD1-positive cell density lack prognostic specificity when assessed outside the broader immune network.

Macrophage-associated markers displayed a different pattern. CD68-positive and CD163-positive macrophage density increased substantially during progression toward invasive SCC, and intraepithelial CD68-positive macrophage density showed a particularly clear control-to-dysplasia-to-SCC gradient. Advanced tumors also contained higher intraepithelial CD68-positive and CD163-positive macrophage density than early-stage tumors, whereas stromal macrophage density were similar between stage groups. These findings suggest that progression may involve not only increasing macrophage density but also their redistribution toward tumor epithelial nests, where macrophages may interact more directly with malignant cells. The strong relationship between FOXP3/CTLA4 double-positive cell density and CD68-positive macrophage density in high-grade dysplasia further supports coordinated regulatory and macrophage-associated remodeling, although functional studies would be required to establish direct interactions.

The adverse association between higher intraepithelial CD68-positive macrophage density and DSS is supported by two recent LSCC studies. Tudor et al. reported that higher intratumoral CD68-positive and CD163-positive macrophage density were associated with shorter DSS and that intratumoral CD68-positive macrophage densities predicted recurrence [22]. Rzepakowska et al. similarly found that higher CD68-positive macrophage densities in the tumor epithelial compartment were associated with shorter DFS in advanced LSCC, while higher stromal CD68-positive macrophage densities were associated with poorer overall survival [35]. In our cohort, CD163-positive macrophage densities were not associated with survival, while CD68-positive macrophage densities were related to DSS but not DFS. Differences in cohort composition, treatment, scoring, cut-offs, and outcome definitions may account for these discrepancies. Because CD68 and CD163 are broad phenotypic markers that do not capture the full continuum of macrophage states, our findings should not be interpreted as evidence that all CD68-positive macrophages are uniformly protumoral. Rather, a high macrophage burden within the epithelial tumor compartment appears to identify a subgroup with more aggressive biological behavior.

The favorable prognostic associations of FOXP3-positive and CTLA4-positive cell densities were initially counterintuitive because both markers are conventionally linked to regulatory T-cell activity and immune suppression [36,37]. In our cohort, however, higher intraepithelial FOXP3-positive cell densities and higher stromal CTLA4-positive cell densities were associated with reduced risks of recurrence and disease-specific death, while higher intraepithelial FOXP3/CTLA4 double-positive cell densities were associated with longer DFS. These associations were particularly pronounced in advanced-stage LSCC.

The prognostic significance of FOXP3-positive cell densities is known to vary across tumor types and spatial contexts. In a meta-analysis of 76 studies, Shang et al. reported favorable associations in several tumor groups, including head and neck malignancies, despite an adverse overall effect across cancers [38]. In the present cohort, the strong relationships among FOXP3-positive, CTLA4-positive, PD1-positive, CD4-positive, and CD8-positive cell densities suggest that FOXP3 and CTLA4 were components of a coordinated immune-inflamed microenvironment rather than isolated indicators of immune suppression. Their favorable prognostic associations may therefore reflect the presence of a more substantial and organized underlying antitumor immune response, accompanied by compensatory regulatory mechanisms. The compartment-specific direction of the results is particularly informative: intraepithelial FOXP3-positive and stromal CTLA4-positive cell densities, rather than all measurements of these markers, carried the clearest prognostic information. These observational findings do not establish a direct protective function of FOXP3 or CTLA4 but reinforce the conclusion that their biological meaning depends on the spatial immune context in which they are detected.

The Protective Immune Score (PIS) was developed to translate this compartment-specific prognostic pattern into a simple model. One point was assigned for high intraepithelial FOXP3-positive cell density and one point for high stromal CTLA4-positive cell density. The score remained independently associated with DSS (adjusted HR 0.29, 95% CI 0.16–0.55) and DFS (adjusted HR 0.29, 95% CI 0.17–0.52) after adjustment for advanced stage and nodal status. These findings suggest that combining biologically complementary variables from different tissue compartments may capture prognostic information not represented by conventional staging or either marker alone. The PIS is intentionally simple, using only two routinely assessed immunohistochemical markers, which may facilitate its validation and reproducibility in independent cohorts. The broader principle of integrating multiple immune parameters into composite scores is supported by previous studies. The internationally validated consensus Immunoscore combines CD3-positive and CD8-positive cell densities across spatial tumor compartments and provides prognostic information beyond conventional staging in colon cancer [39]. Other models have incorporated the balance between antitumor and tumor-supportive immune populations. A signature of immune activation based on the ratio of CD8-positive lymphocytes to the combined density of CD8-positive lymphocytes and CD68-positive/CD163-positive macrophages showed independent prognostic relevance across several solid tumors [40]. In LSCC, Zhang et al. developed a cytotoxic T-lymphocyte-to-immunosuppressive-cell ratio integrating CD8-positive cells, M2-like macrophages, and N2-like neutrophils, which demonstrated stronger prognostic performance than a conventional CD3/CD8-based Immunoscore in their cohorts [41]. A further LSCC-specific comparison is provided by the immunophenotypic classification proposed by Sirbu et al. [42]. In their cohort of 82 LSCCs, tumors were categorized as active, mixed, or immunosuppressive according to semiquantitative evaluation of multiple immune-cell markers in intratumoral and stromal regions. The active immunotype was associated with better overall survival, consistent with the broader concept that coordinated immune-rich configurations may indicate favorable outcomes. However, the approaches are not directly interchangeable. Their classification integrates a broader lineage-marker panel using descriptive categories, whereas the PIS applies a transparent additive rule to two quantitatively dichotomized, compartment-specific variables selected through survival analyses. This simplicity does not establish superiority, and both approaches require external validation, evaluation of interobserver reproducibility, and comparison with established clinicopathological models. Although these models differ in marker selection and scoring methodology, they support the concept that composite measures may better represent immune organization than isolated biomarkers. The PIS is not intended to establish superiority over these approaches and should currently be regarded as a proof-of-concept prognostic score requiring external validation. No single biomarker has adequately captured the complexity of antitumor immunity in HNSCC, and the individual performance of PD-L1, immune-cell densities, tumor mutational burden, HPV status, and immune-gene signatures remains limited [34,43]. Consistent with this, PD1-positive cell densities alone were not prognostic in our cohort, whereas the spatially informed combination of intraepithelial FOXP3-positive and stromal CTLA4-positive cell densities retained independent prognostic value. However, the PIS cannot be considered predictive of response to immunotherapy because patients in this cohort did not receive PD1/PD-L1- or CTLA4-directed treatment. Although acquired resistance to immune checkpoint inhibition is common across tumor types [44], the present study cannot link the identified immune configurations to treatment response or resistance. Future studies may validate the PIS in independent cohorts and determine whether more detailed spatial and functional analyses improve its prognostic or potential predictive performance.

The present study has several strengths. It evaluates a broad immune panel across benign lesions, dysplasia, and invasive carcinoma rather than restricting the analysis to established LSCC. It distinguishes intraepithelial, stromal, and overall cell densities, integrates quantitative findings with correlation networks, and relates compartment-specific immune patterns to both DFS and DSS. The inclusion of dysplasia provides insight into when immune alterations emerge, while the correlation analysis demonstrates that immune remodeling involves coordinated lymphoid, regulatory, checkpoint-associated, and macrophage populations rather than isolated changes in individual markers. Separate early- and advanced-stage analyses further show that the prognostic significance of immune contexture may be particularly evident in biologically aggressive disease.

Several limitations must also be acknowledged. First, this was a retrospective single-center study, and selection bias and unmeasured confounding cannot be excluded. Second, tissue microarray analysis may not fully capture intratumoral heterogeneity despite compartment-specific evaluation and the use of serial sections and representative tissue cores. Core loss or insufficient tissue resulted in variable numbers of evaluable samples across markers. Third, only a small number of dysplastic lesions progressed to SCC; the observed associations involving stromal FOXP3-positive density and intraepithelial FOXP3/CTLA4 double-positive cell density should therefore be considered hypothesis-generating rather than validated predictors of malignant transformation. Similarly, the relatively small numbers of recurrences and disease-specific deaths reduced statistical precision and may have limited the reliability of some subgroup and cut-off analyses. Fourth, the PIS was developed and evaluated in the same dataset and requires validation in independent, preferably multicenter cohorts with prespecified cut-offs. Fifth, immunohistochemical markers are phenotypic surrogates rather than direct measures of function. FOXP3 positivity does not identify all regulatory T-cell states with complete specificity, PD1 positivity does not distinguish activation from terminal exhaustion, and CD68-positive and CD163-positive macrophage density provide only a simplified representation of macrophage diversity. Finally, because the cohort was not treated with immune checkpoint inhibitors, no conclusions can be drawn regarding prediction of therapeutic response or resistance.

In summary, laryngeal carcinogenesis was characterized by progressive, compartment-specific, and coordinated reorganization of the immune microenvironment. Early intraepithelial T-cell and checkpoint-associated changes were already detectable in dysplasia, whereas invasive SCC was associated with extensive stromal accumulation and increasingly interconnected networks involving effector T cells, regulatory populations, checkpoint-associated cells, and macrophages. The predominance of these correlations in the stroma identifies this compartment as the principal site of coordinated immune organization and represents a central biological finding of the study. Within this spatial context, higher intraepithelial FOXP3-positive cell density and higher stromal CTLA4-positive cell density were associated with favorable clinical outcomes and formed a PIS with independent prognostic value for DFS and DSS. In contrast, higher intraepithelial CD68-positive macrophage density identified a more aggressive phenotype. Collectively, the results support a model in which LSCC behavior is determined not only by the quantity of immune infiltration but also by its spatial distribution, intercellular coordination, and balance between immune activation and immune regulation.

## 5. Conclusions

Our findings demonstrate that laryngeal carcinogenesis is accompanied by progressive and spatially coordinated remodeling of the immune microenvironment, beginning in dysplastic lesions and becoming most pronounced in invasive LSCC. The simultaneous increase in effector, regulatory, checkpoint, and macrophage-associated markers indicates that LSCC develops an immune-inflamed but concurrently immunoregulatory microenvironment rather than a purely immunosuppressive one. High intratumoral FOXP3 density and stromal CTLA4 cell density were associated with improved disease-free and disease-specific survival, whereas high intratumoral CD68 cell density identified a subgroup with poorer disease-specific outcome. Importantly, the Protective Immune Score combining intratumoral FOXP3 and stromal CTLA4 remained independently associated with reduced risks of recurrence and disease-specific death, supporting the potential value of spatially defined composite immune biomarkers.

These results should be interpreted in the context of the retrospective single-center design, tissue microarray–related sampling limitations, and the relatively small numbers of dysplastic lesions progressing to carcinoma, recurrences, and disease-specific deaths. The Protective Immune Score therefore requires validation in larger, independent prospective cohorts. Further studies should also determine whether the spatial immune patterns identified in this study have predictive relevance for response to immune checkpoint inhibition.

## Figures and Tables

**Figure 1:**
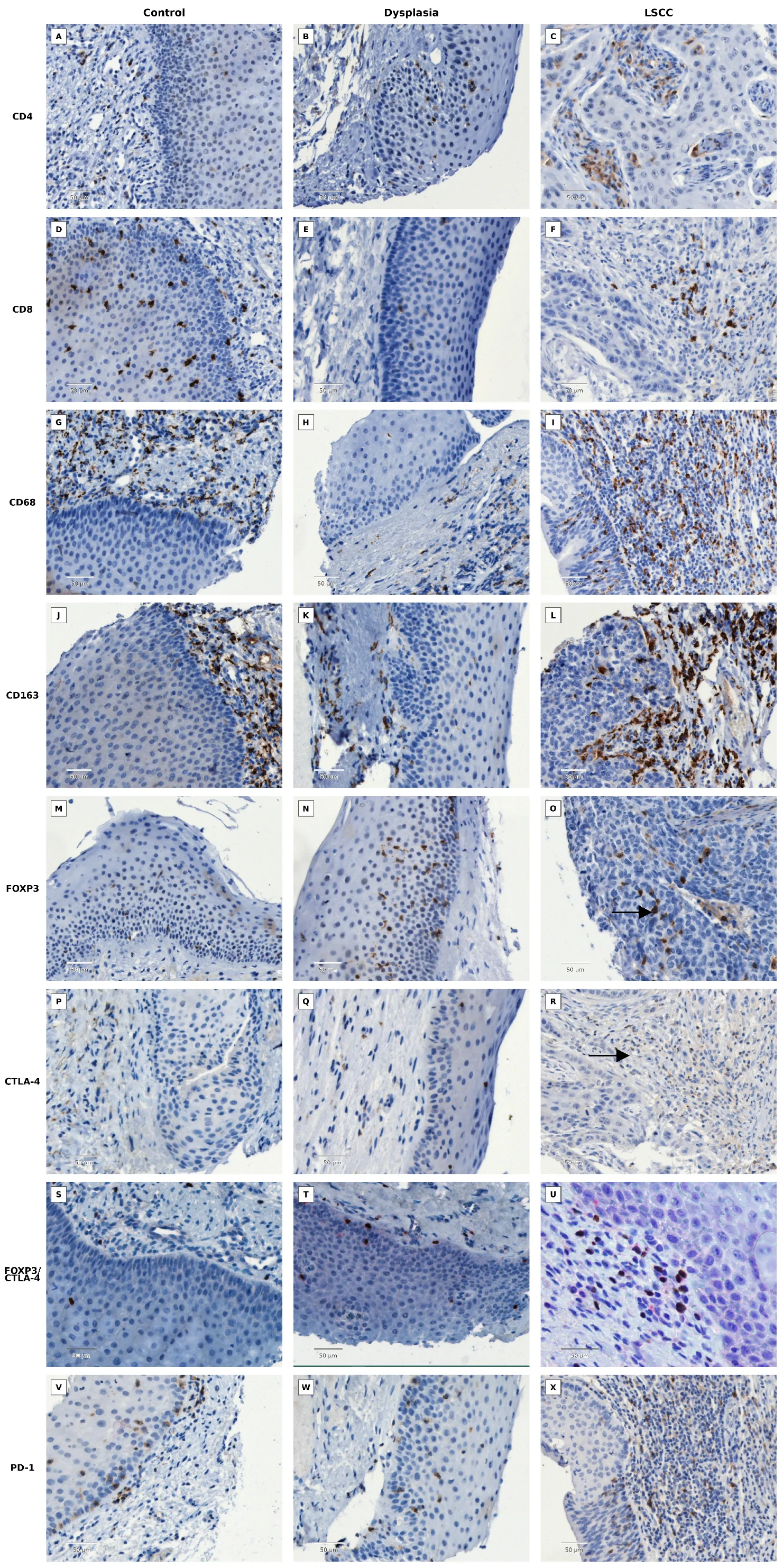
Representative immunohistochemical staining patterns of immune markers across laryngeal carcinogenesis. Representative staining for CD4 (**A**–**C**), CD8 (**D**–**F**), CD68 (**G**–**I**), CD163 (**J**–**L**), FOXP3 (**M**–**O**), CTLA-4 (**P**–**R**), FOXP3/CTLA-4 double immunostaining (**S**–**U**), and PD-1 (**V**–**X**) is shown in benign laryngeal lesions (control; left column), laryngeal epithelial dysplasia (middle column), and invasive laryngeal squamous cell carcinoma (LSCC; right column). Arrows indicate intraepithelial FOXP3-positive cells and stromal CTLA-4-positive cells. In the FOXP3/CTLA-4 double immunohistochemistry, nuclear FOXP3 positivity is visualized in brown, whereas cytoplasmic CTLA-4 positivity is visualized in red. Representative LSCC panels illustrate the compartment-specific distribution of immune cells within tumor nests and the surrounding stroma. Original magnification: ×200 for all panels except panel U, which is shown at ×400 to provide a clearer visualization of the double-staining pattern and the distinct nuclear FOXP3 and cytoplasmic CTLA-4 localization. Scale bars are shown in each panel.

**Figure 2:**
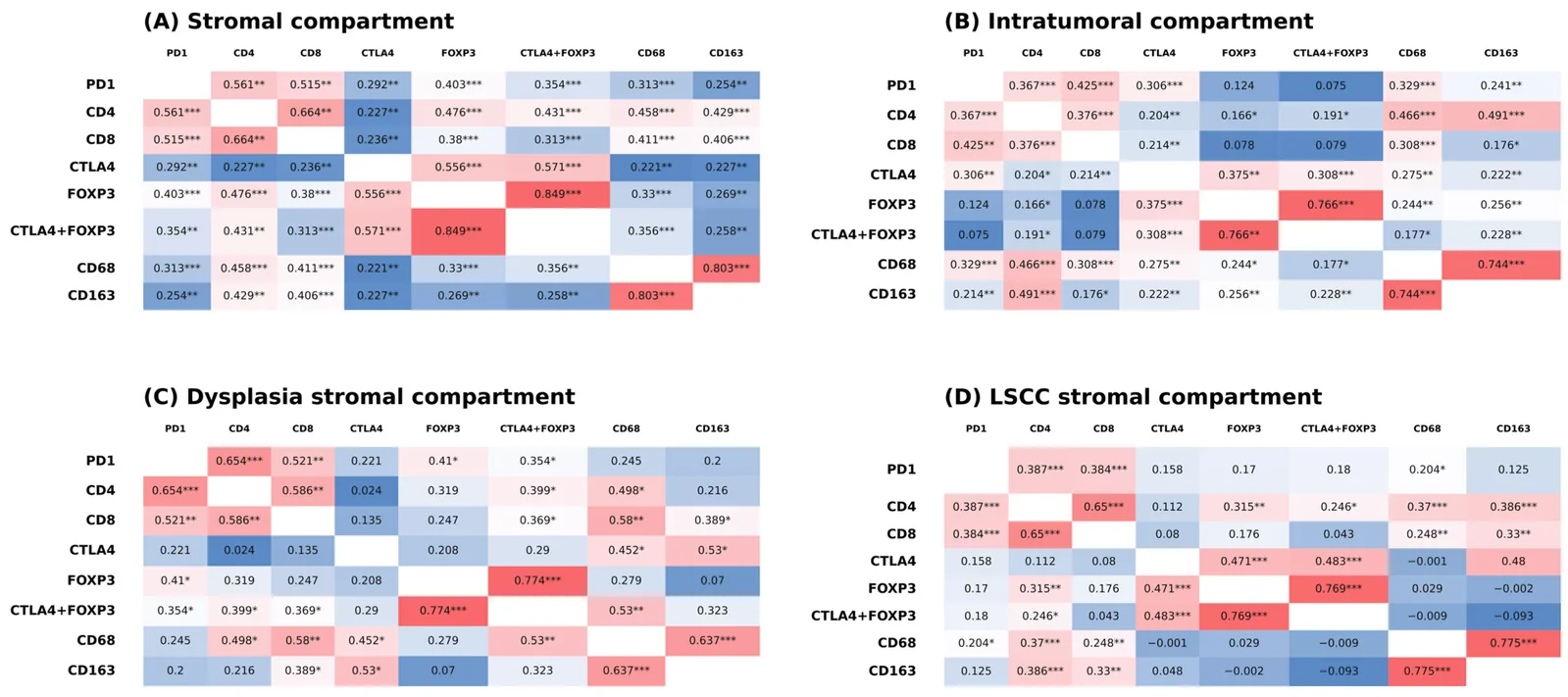
Correlation heatmaps illustrating compartment-specific immune correlation networks during laryngeal carcinogenesis. Spearman correlation coefficients (rs) between immune cell density and immune checkpoint markers are shown for (**A**) all samples–stromal compartment, (**B**) all samples–intraepithelial/intratumoral compartment, (**C**) dysplasia stromal compartment, and (**D**) LSCC stromal compartment. Red indicates positive correlations and blue indicates negative correlations; color intensity reflects the strength of the correlation. Statistically significant correlations are indicated by asterisks (* *p* < 0.05, ** *p* < 0.01, *** *p* < 0.001).

**Figure 3:**
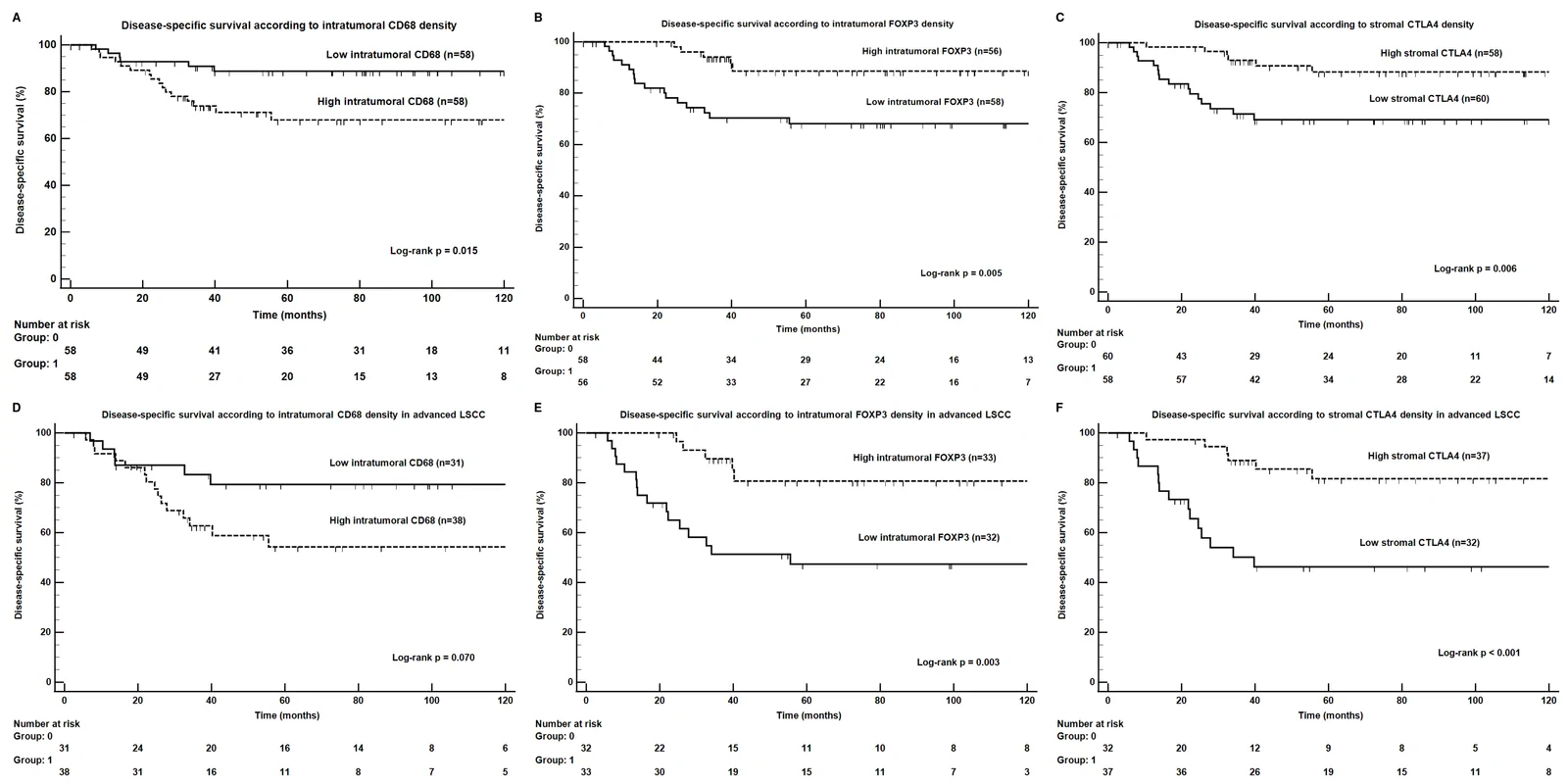
Disease-specific survival according to intratumoral CD68, intratumoral FOXP3, and stromal CTLA-4 immune-cell density in patients with LSCC. Kaplan–Meier analysis of disease-specific survival (DSS) according to intratumoral CD68-positive cell density (**A**), intratumoral FOXP3-positive cell density (**B**), and stromal CTLA-4-positive cell density (**C**) in the overall LSCC cohort. Corresponding analyses restricted to patients with advanced-stage LSCC are shown for intratumoral CD68-positive cell density (**D**), intratumoral FOXP3-positive cell density (**E**), and stromal CTLA-4-positive cell density (**F**). Patients were dichotomized into low- and high-density groups using the respective median value for each immune marker as the cut-off. Survival curves were compared using the log-rank test. Numbers at risk are shown below each Kaplan–Meier plot. Curves are graphically truncated at 120 months; all available follow-up data were retained in the survival analyses.

**Figure 4:**
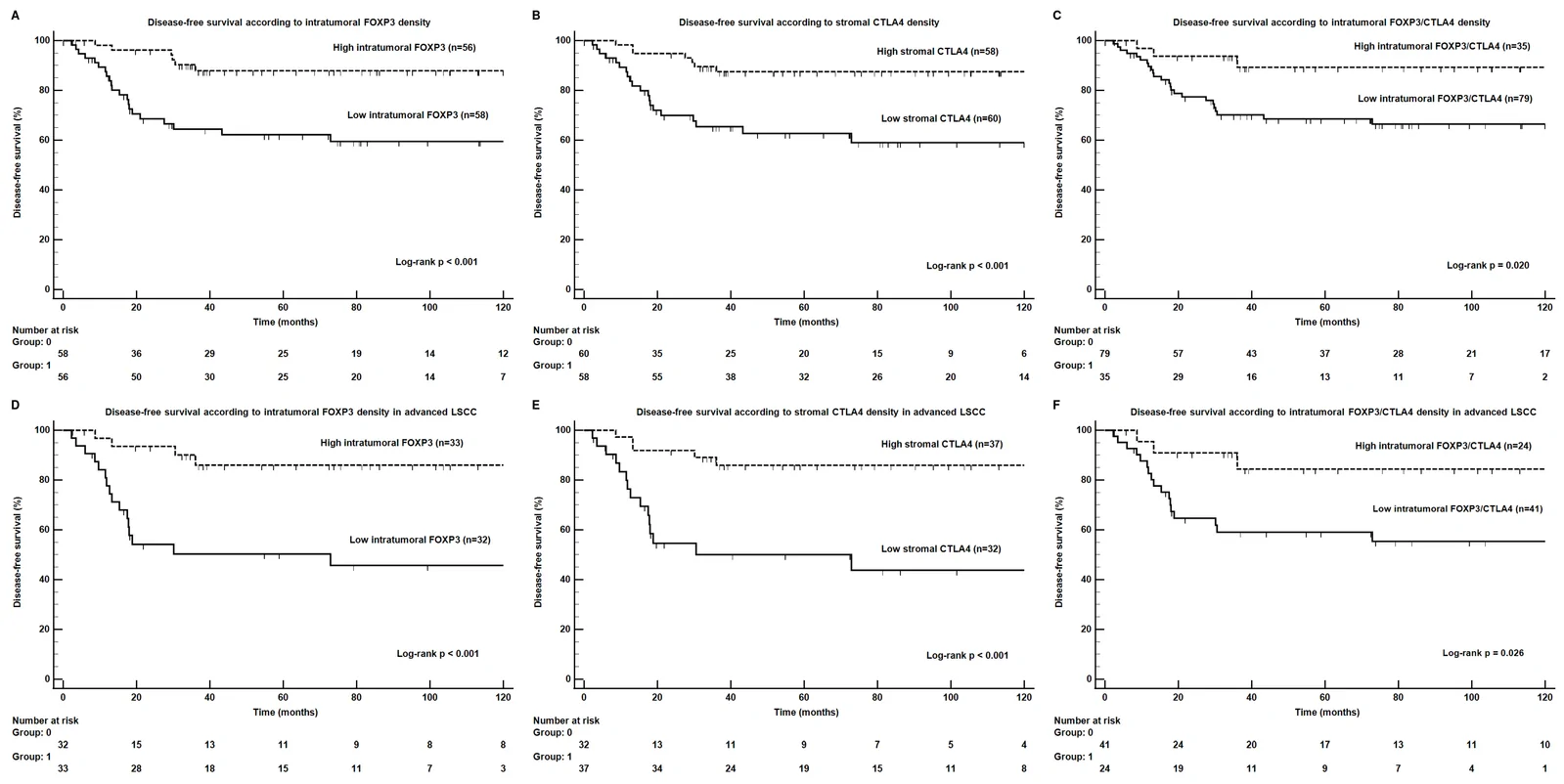
Disease-free survival according to intratumoral FOXP3, stromal CTLA4, and intratumoral FOXP3/CTLA4 double-positive cell density in patients with LSCC. Kaplan–Meier analysis of disease-free survival (DFS) according to intratumoral FOXP3-positive cell density (**A**), stromal CTLA4-positive cell density (**B**), and intratumoral FOXP3/CTLA4 double-positive cell density (**C**) in the overall LSCC cohort. Corresponding analyses restricted to patients with advanced-stage LSCC are shown for intratumoral FOXP3-positive cell density (**D**), stromal CTLA4-positive cell density (**E**), and intratumoral FOXP3/CTLA4 double-positive cell density (**F**). Patients were dichotomized into low- and high-density groups using the respective median value for each immune marker as the cut-off. Survival curves were compared using the log-rank test. Numbers at risk are shown below each Kaplan–Meier plot. Curves are graphically truncated at 120 months; all available follow-up data were retained in the survival analyses.

**Figure 5:**
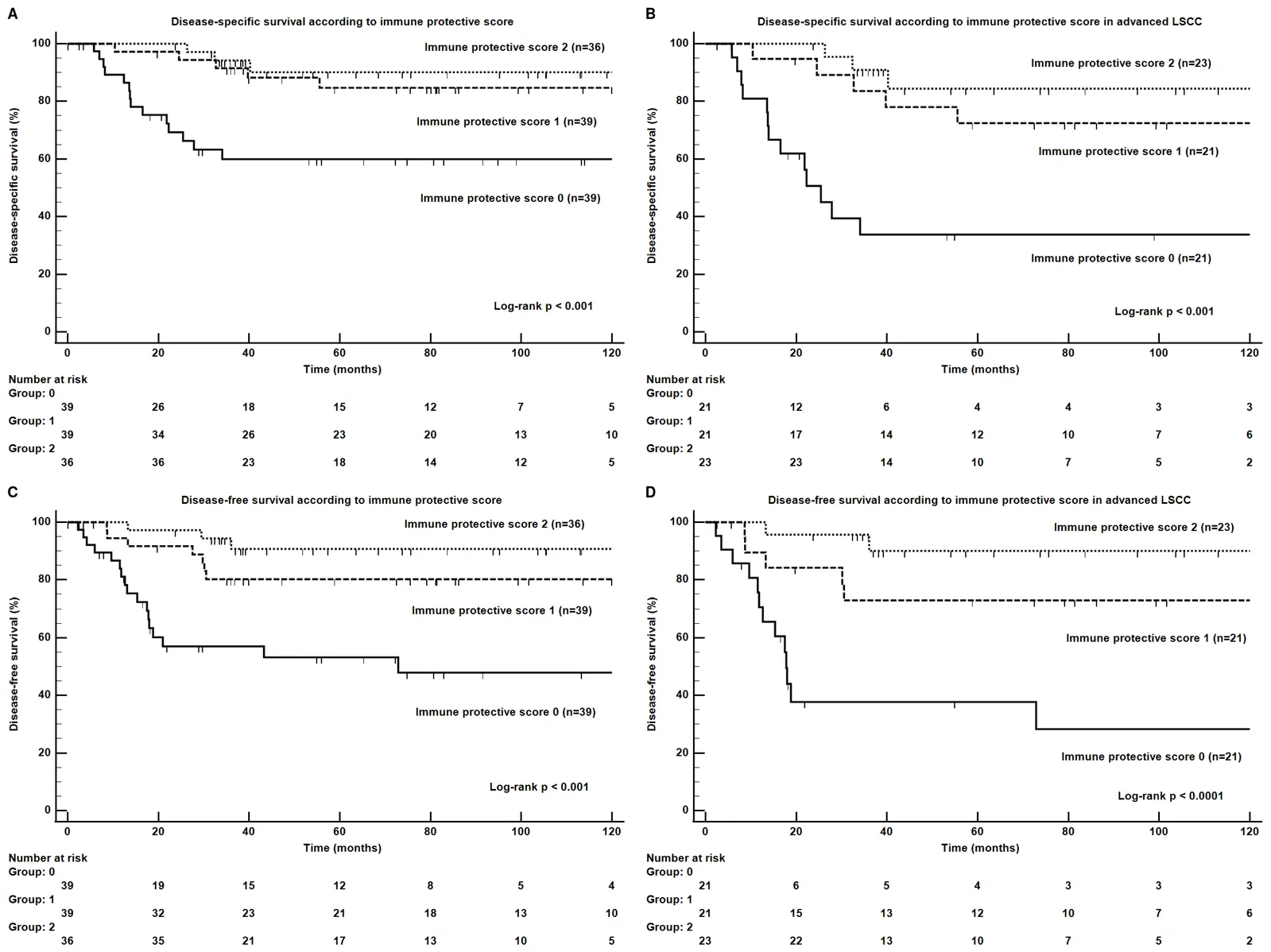
Prognostic significance of the Protective Immune Score in patients with LSCC. Kaplan–Meier analysis of disease-specific survival (DSS) according to the Protective Immune Score (PIS; 0–2) in the overall LSCC cohort (**A**) and in patients with advanced-stage LSCC (**B**), and disease-free survival (DFS) in the overall LSCC cohort (**C**) and in patients with advanced-stage LSCC (**D**). The PIS was constructed by assigning one point each for high intratumoral FOXP3-positive cell density and high stromal CTLA4-positive cell density, resulting in scores ranging from 0 to 2. Survival curves were compared using the log-rank test. Numbers at risk are shown below each Kaplan–Meier plot. Curves are graphically truncated at 120 months; all available follow-up data were retained in the survival analyses.

**Table 1:** Clinicopathological characteristics of the 127 patients with laryngeal squamous cell carcinoma (LSCC).

Variable	Category	LSCC (N = 127)
Patient characteristics		
Age, years	≤60	45 (35.4)
	>60	82 (64.6)
Sex	Female	10 (7.9)
	Male	117 (92.1)
Alcohol consumption	No	64 (50.4)
	Yes	63 (49.6)
Smoking	No	28 (22.0)
	Yes	99 (78.0)
Tumor characteristics		
pT classification	T1	33 (26.0)
	T2	22 (17.3)
	T3	48 (37.8)
	T4	24 (18.9)
pN classification	N0	100 (78.7)
	N1	15 (11.8)
	N2	10 (7.9)
	N3	2 (1.6)
M classification	M0	124 (97.6)
	M1	3 (2.4)
UICC stage	I	33 (26.0)
	II	22 (17.3)
	III	40 (31.5)
	IV	32 (25.2)
Disease stage	Early-stage LSCC	55 (43.3)
	Advanced-stage LSCC	72 (56.7)
Lymph node status	Negative	100 (78.7)
	Positive	27 (21.3)
Histological grade	G1	31 (24.4)
	G2	61 (48.0)
	G3	23 (18.1)
	NA	12 (9.4)
Lymphovascular invasion	No	58 (45.7)
	Yes	45 (35.4)
	NA	24 (18.9)
Perineural invasion	No	87 (68.5)
	Yes	15 (11.8)
	NA	25 (19.7)
Treatment		
Neck dissection	No	90 (70.9)
	Yes	37 (29.1)
Postoperative treatment	None	59 (46.5)
	PORT	67 (52.8)
	Radiotherapy only	1 (0.8)
Clinical outcomes		
Recurrence	No	98 (77.2)
	Yes	29 (22.8)
Pattern of recurrence	None	98 (77.2)
	Local	15 (11.8)
	Regional	7 (5.5)
	Distant	7 (5.5)
Time to recurrence, months	Median (range)	17.5 (2.3–72.9)
Patient outcome	Alive at last follow-up	69 (54.3)
	Died of LSCC	23 (18.1)
	Died of other causes	35 (27.6)
Follow-up duration, months	Median (reverse Kaplan–Meier)	113.3 (95% CI, 91.5–128.0)
Second primary malignancy		
Occurrence	No	96 (75.6)
	Yes	31 (24.4)
Site	Head and neck	8 (25.8)
	Other sites	23 (74.2)
Time to second primary malignancy, months	Median (range)	62.0 (0.2–155.2)

Note: Data are presented as *n* (%) unless otherwise indicated. Continuous variables are presented as median (range), except follow-up duration, which is reported as the median estimated using the reverse Kaplan–Meier method with its 95% confidence interval (CI). Abbreviations: LSCC, laryngeal squamous cell carcinoma; PORT, postoperative radiotherapy; NA, data not available (missing data); CI, confidence interval.

**Table 2:** Distribution of immune cell densities across normal mucosa, dysplasia and invasive LSCC according to tissue compartment.

Variable		Control, Median (IQR), *n*	Dysplasia, Median (IQR), *n*	SCC, Median (IQR), *n*	Overall *p* Value	Significant Post Hoc Comparisons
T-cell markers						
Intraepithelial compartment	CD4	0.5 (0–1.77), *n* = 37	1.1 (0.2–3.0), *n* = 40	1.5 (0.5–2.85), *n* = 113	0.005	Dysplasia > control; SCC > control
	CD8	1.0 (0.25–4.75), *n* = 36	2.0 (1.13–5.25), *n* = 39	2.5 (1.33–5.78), *n* = 115	0.008	Dysplasia > control; SCC > control
Stromal compartment	CD4	1.5 (0–5.04), *n* = 37	1.75 (0.5–5.0), *n* = 40	9.0 (5.0–15.0), *n* = 113	<0.0001	SCC > control, SCC > dysplasia
	CD8	1.9 (0.5–4.0), *n* = 36	2.0 (0.63–5.50), *n* = 39	6.67 (2.63–13.88), *n* = 115	<0.0001	SCC > control, SCC > dysplasia
Combined compartments (overall)	CD4	1.5 (0.08–4.13), *n* = 37	2.3 (1.0–5.0), *n* = 40	8.0 (5.0–18.1), *n* = 113	<0.0001	SCC > control, SCC > dysplasia
	CD8	2.75 (1.0–4.7), *n* = 36	4.0 (2.0–6.83), *n* = 39	8.0 (3.7–20.0), *n* = 115	<0.0001	SCC > control, SCC > dysplasia
Macrophage markers						
Intraepithelial compartment	CD68	0 (0–0.63), *n* = 33	1.0 (0–3.0), *n* = 41	2.9 (1.4–5.2), *n* = 116	<0.0001	Control < dysplasia < SCC
	CD163	0 (0–1.0), *n* = 32	0.3 (0–1.0), *n* = 40	2.5 (0.8–5.0), *n* = 119	<0.0001	SCC > control, SCC > dysplasia
Stromal compartment	CD68	1.0 (0–4.25), *n* = 33	2.5 (0–5.0), *n* = 41	7.0 (3.15–10.1), *n* = 116	<0.0001	SCC > control, SCC > dysplasia
	CD163	1.0 (0–5.0), *n* = 32	4.25 (1.25–6.25), *n* = 40	10.0 (4.17–19.63), *n* = 119	<0.0001	SCC > control, SCC > dysplasia
Combined compartments (overall)	CD68	2.0 (0.38–5.0), *n* = 33	4.0 (0.95–5.63), *n* = 41	9.5 (5.9–15.0), *n* = 116	<0.0001	SCC > control, SCC > dysplasia
	CD163	3.1 (0.65–6.5), *n* = 32	4.5 (1.5–6.5), *n* = 40	15.0 (7.0–25.0), *n* = 119	<0.0001	SCC > control, SCC > dysplasia
Checkpoint/regulatory markers						
Intraepithelial compartment	CTLA4	0 (0–0.44), *n* = 31	0.5 (0–1.0), *n* = 38	1.0 (0–1.63), *n* = 118	0.0002	Dysplasia > control; SCC > control
	FOXP3	0 (0–0.5), *n* = 31	0 (0–0.75), *n* = 42	0.17 (0–0.9), *n* = 114	0.535	
	FOXP3/CTLA4	0 (0–0.5), *n* = 30	0 (0–0.5), *n* = 42	0 (0–0.33), *n* = 114	0.793	
	PD1	0.09 (0–2.0), *n* = 38	1.75 (0.5–3.0), *n* = 38	2.0 (1.0–3.0), *n* = 115	<0.0001	Dysplasia > control; SCC > control
Stromal compartment	CTLA4	0.75 (0–5.0), *n* = 30	0.5 (0–1.0), *n* = 38	4.0 (0.75–7.25), *n* = 118	<0.0001	SCC > control, SCC > dysplasia
	FOXP3	0.5 (0–1.0), *n* = 31	0.5 (0–1.0), *n* = 42	3.5 (1.0–6.67), *n* = 114	<0.0001	SCC > control, SCC > dysplasia
	FOXP3/CTLA4	0 (0–0.5), *n* = 31	0 (0–0.5), *n* = 42	1.0 (0.5–2.5), *n* = 114	<0.0001	SCC > control, SCC > dysplasia
	PD1	1.0 (0–1.92), *n* = 35	2.0 (1.0–4.92), *n* = 35	4.42 (2.67–7.0), *n* = 114	<0.0001	Control < dysplasia < SCC
Combined compartments (overall)	CTLA4	1.0 (0–4.0), *n* = 31	0.5 (0–1.0), *n* = 38	3.0 (0.5–5.0), *n* = 118	<0.0001	SCC > control, SCC > dysplasia
	FOXP3	0.5 (0–1.0), *n* = 31	0.2 (0–1.0), *n* = 42	3.0 (1.0–5.0), *n* = 114	<0.0001	SCC > control, SCC > dysplasia
	FOXP3/CTLA4	0 (0–0.5), *n* = 31	0.2 (0–1.0), *n* = 42	0.9 (0.4–2.0), *n* = 114	<0.0001	SCC > control, SCC > dysplasia
	PD1	0.5 (0–1.5), *n* = 38	2.25 (1.0–3.0), *n* = 38	3.0 (2.0–5.0), *n* = 115	<0.0001	Control < dysplasia < SCC

Note: Data are presented as median (interquartile range, IQR). Comparisons among groups were performed using the Kruskal–Wallis test followed, when appropriate, by Conover’s post hoc test. Only statistically significant pairwise comparisons are shown in the final column. The number of evaluable cases varied across immune markers because of tissue microarray core loss or insufficient tissue for analysis. Immune cell densities are expressed as the percentage (%) of the respective tissue compartment occupied by immunohistochemically positive immune cells.

**Table 3:** Immune cell densities according to dysplasia grade and progression to SCC.

Variable	Low-Grade Dysplasia, Median (IQR), *n*	High-Grade Dysplasia, Median (IQR), *n*	*p* Value	Non-Progressors, Median (IQR), *n*	Progression to SCC, Median (IQR), *n*	*p* Value
T-cell markers						
CD4 stromal	1.0 (0.15–2.0), *n* = 22	3.7 (1.0–6.5), *n* = 18	**0.012**	1.5 (0.5–4.45), *n* = 35	7.5 (1.75–17.50), *n* = 5	**0.039**
CD4 overall	1.5 (0.6–3.0), *n* = 22	4.28 (2.0–7.5), *n* = 18	**0.016**	2.0 (0.7–4.29), *n* = 35	7.5 (2.5–9.75), *n* = 5	0.093
CD8 stromal	2.25 (0.4–4.75), *n* = 20	2.0 (1.13–7.04), *n* = 19	0.284	2.0 (0.5–5.0), *n* = 34	5.67 (3.5–11.75), *n* = 5	0.084
CD8 overall	3.5 (1.5–6.5), *n* = 20	4.0 (2.1–8.7)	0.367	3.5 (2.0–6.3), *n* = 34	5.67 (4.25–14.0), *n* = 5	0.178
Macrophage markers						
CD68 intraepithelial	0.4 (0–1.16), *n* = 23	1.25 (0.8–3.33), *n* = 18	0.051	1.0 (0.13–3.0), *n* = 36	0.9 (0.8–1.96), *n* = 5	0.658
CD68 stromal	1.75 (0–4.75), *n* = 23	4.0 (1.0–6.0), *n* = 18	0.150	2.38 (0–5.0), *n* = 36	7.5 (1.32–9.75), *n* = 5	0.106
CD68 overall	2.0 (0.05–4.38), *n* = 23	5.0 (2.5–6.5), *n* = 18	**0.011**	3.5 (0.9–5.25), *n* = 36	5.0 (1.69–10.88), *n* = 5	0.327
Checkpoint/regulatory markers						
FOXP3 intraepithelial	0 (0–0.5), *n* = 22	0 (0–1.34), *n* = 20	0.281	0 (0–0.63), *n* = 36	0.75 (0–1.67), *n* = 6	0.114
FOXP3 stromal	0.13 (0–0.5), *n* = 22	0.88 (0–3.34), *n* = 20	0.052	0.25 (0–0.88), *n* = 36	1.84 (1.0–4.5), *n* = 6	**0.030**
FOXP3 overall	0.2 (0–0.5), *n* = 22	0.88 (0–2.75), *n* = 20	**0.041**	0.2 (0–0.73), *n* = 36	1.75 (1.0–3.0), *n* = 6	**0.013**
FOXP3/CTLA4 intraepithelial	0 (0–0.5), *n* = 22	0 (0–0.88), *n* = 20	0.558	0 (0–0.38), *n* = 36	0.75 (0–1.33), *n* = 6	**0.037**
FOXP3/CTLA4 stromal	0 (0–0.25), *n* = 22	0.13 (0–1.75), *n* = 20	**0.039**	0 (0–0.5), *n* = 36	1.0 (0–3.0), *n* = 6	0.181
FOXP3/CTLA4 overall	0 (0–0.2), *n* = 22	0.5 (0–1.0), *n* = 20	**0.045**	0.1 (0–0.5), *n* = 36	0.75 (0.2–2.0), *n* = 6	0.072
PD1 intraepithelial	1.0 (0–2.38), *n* = 19	3.0 (1.32–5.0), *n* = 19	**0.005**	1.5 (0.5–3.0), *n* = 34	3.5 (1.25–5.34), *n* = 4	0.270
PD1 stromal	1.0 (0.75–2.25), *n* = 19	4.34 (2.0–6.0), *n* = 19	**0.004**	2.0 (0.87–4.0), *n* = 34	4.84 (3.34–6.75), *n* = 4	0.106
PD1 overall	1.0 (0.5–1.88), *n* = 19	3.0 (2.5–5.0), *n* = 19	**0.001**	2.0 (1.0–3.0), *n* = 34	4.25 (2.5–5.75), *n* = 4	0.085

Note: Data are presented as median (interquartile range, IQR). Group comparisons were performed using the Mann–Whitney U test. Due to tissue microarray core loss or insufficient tissue, the number of evaluable cases varied across immune markers. Statistically significant *p* values are shown in bold. Immune cell densities are expressed as the percentage (%) of the respective tissue compartment occupied by immunohistochemically positive immune cells.

**Table 4:** Immune cell densities in early- and advanced-stage SCC.

Variable	Early-Stage SCC, Median (IQR), *n*	Advanced-Stage SCC, Median (IQR), *n*	*p* Value
T-cell markers			
CD4 intraepithelial	1.8 (0.65–2.78), *n* = 45	1.38 (0.45–2.90), *n* = 68	0.683
CD4 stromal	7.5 (4.92–15.0), *n* = 45	9.71 (5.85–16.45), *n* = 68	0.156
CD8 intraepithelial	2.5 (1.0–4.95), *n* = 47	3.0 (1.4–6.4), *n* = 68	0.434
CD8 stromal	5.0 (2.0–9.75), *n* = 47	9.0 (4.5–15.0), *n* = 68	**0.010**
Macrophage markers			
CD68 intraepithelial	2.0 (1.0–4.0), *n* = 47	3.3 (1.5–6.13), *n* = 69	**0.028**
CD68 stromal	6.5 (4.0–10.0), *n* = 47	7.0 (2.88–11.08), *n* = 69	0.948
CD163 intraepithelial	2.0 (0.4–3.67), *n* = 48	3.0 (1.0–6.83), *n* = 71	**0.032**
CD163 stromal	10.0 (6.3–17.1), *n* = 48	11.0 (2.33–20.0), *n* = 71	0.970
Checkpoint/regulatory markers			
FOXP3 intraepithelial	0 (0–0.73), *n* = 49	0.2 (0–0.96), *n* = 65	0.544
FOXP3 stromal	2.0 (0.5–5.0), *n* = 49	4.0 (1.94–7.33), *n* = 65	**0.006**
FOXP3/CTLA4 intraepithelial	0 (0–0), *n* = 49	0 (0–0.5), *n* = 65	0.113
FOXP3/CTLA4 stromal	0.83 (0.25–2.0), *n* = 49	1.0 (0.5–2.66), *n* = 65	0.104
PD1 intraepithelial	2.0 (1.0–3.0), *n* = 48	2.1 (1.0–3.11), *n* = 67	0.599
PD1 stromal	3.5 (2.37–6.94), *n* = 48	5.0 (3.0–7.38), *n* = 67	0.199

Note: Data are presented as median (interquartile range, IQR). Group comparisons were performed using the Mann–Whitney U test. Due to tissue microarray core loss or insufficient tissue, the number of evaluable cases varied across immune markers. Statistically significant *p* values are shown in bold. Immune cell densities are expressed as the percentage (%) of the respective tissue compartment occupied by immunohistochemically positive immune cells.

**Table 5:** Key compartment-specific immune correlation networks during laryngeal carcinogenesis.

Study Group	Compartment	Marker Pair	N	Spearman r_s_	*p* Value
All samples	Intraepithelial	PD1 ↔ CD4	172	0.367	<0.0001
	Intraepithelial	PD1 ↔ CD8	174	0.425	<0.0001
	Stromal	PD1 ↔ CD4	167	0.561	<0.0001
	Stromal	PD1 ↔ CD8	169	0.515	<0.0001
	Stromal	PD1 ↔ FOXP3	164	0.403	<0.0001
	Stromal	CTLA4 ↔ FOXP3	176	0.556	<0.0001
Dysplasia, All dysplasia	Intraepithelial	CTLA4 ↔ PD1	34	0.433	0.011
	Intraepithelial	FOXP3/CTLA4 ↔ CD163	35	0.414	0.013
	Intraepithelial	PD1 ↔ CD4	33	0.448	0.009
	Stromal	PD1 ↔ CD4	31	0.654	0.0001
	Stromal	PD1 ↔ CD8	30	0.521	0.003
	Stromal	PD1 ↔ FOXP3	32	0.410	0.019
	Stromal	FOXP3/CTLA4 ↔ CD68	36	0.530	0.0009
	Stromal	CTLA4 ↔ CD163	34	0.530	0.001
	Stromal	CTLA4 ↔ CD68	35	0.452	0.006
Low-grade dysplasia	Stromal	PD1 ↔ CD4	16	0.707	0.002
	Stromal	PD1 ↔ CD8	14	0.668	0.009
	Stromal	CTLA4 ↔ CD68	21	0.573	0.007
	Stromal	CTLA4 ↔ CD163	20	0.653	0.002
High-grade dysplasia	Stromal	FOXP3/CTLA4 ↔ CD4	15	0.660	0.008
	Stromal	FOXP3/CTLA4 ↔ CD68	15	0.752	0.001
LSCC, All LSCC	Intraepithelial	CTLA4 ↔ FOXP3	114	0.421	<0.0001
	Stromal	CTLA4 ↔ FOXP3	114	0.471	<0.0001
Early-stage LSCC	Stromal	PD1 ↔ CD8	43	0.436	0.004
	Stromal	CTLA4 ↔ FOXP3	49	0.466	0.007
Advanced-stage LSCC	Intraepithelial	CTLA4 ↔ FOXP3	65	0.460	0.0001
	Stromal	CTLA4 ↔ FOXP3	65	0.486	<0.0001
	Stromal	PD1 ↔ CD4	63	0.456	0.0002

Note: Only the strongest and biologically relevant statistically significant correlations are presented. Correlations were assessed using Spearman’s rank correlation coefficient (r_s_). Marker pairs are shown using bidirectional notation (↔) to indicate positive associations without implying causality. Owing to tissue microarray core loss or insufficient tissue, the number of evaluable samples varied between analyses.

**Table 6:** Univariate Cox proportional hazards regression analysis for disease-specific survival.

Variable	HR	95%CI	*p* Value
Clinical and histological			
Age > 60 years	0.83	0.36–1.93	0.670
Male sex	2.32	0.31–17.21	0.410
Smoking	2.03	0.60–6.82	0.254
Alcohol consumption	1.56	0.67–3.59	0.301
Histological grade G2–G3	3.87	0.90–16.63	0.069
pT3–4	19.71	2.65–146.30	0.004
UICC stage III–IV	19.71	2.65–146.30	0.004
pN+	3.30	1.44–7.52	0.005
LVI positive	3.34	1.26–8.80	0.015
PNI positive	2.46	0.89–6.85	0.084
Postoperative radiotherapy (PORT)	3.39	1.26–9.13	0.016
Biomarkers			
CD68 intratumoral high	3.03	1.18–7.77	0.021
CD68 stromal high	1.31	0.56–3.02	0.534
CD163 intratumoral high	1.70	0.74–3.88	0.209
CD163 stromal high	1.63	0.71–3.71	0.248
FOXP3 intratumoral high	0.26	0.10–0.72	0.009
FOXP3 stromal high	0.60	0.25–1.43	0.251
CTLA4 intratumoral high	0.63	0.25–1.62	0.343
CTLA4 stromal high	0.29	0.11–0.74	0.010
FOXP3/CTLA4 intratumoral high	0.51	0.17–1.34	0.218
FOXP3/CTLA4 stromal high	0.55	0.22–1.34	0.187
PD1 intratumoral high	1.13	0.50–2.55	0.777
PD1 stromal high	0.98	0.43–2.22	0.958

Note: Univariate Cox proportional hazards regression analysis was performed for disease-specific survival. A total of 23 disease-specific death events occurred in the complete LSCC cohort; the number of evaluable cases and events varied across individual biomarker analyses because of TMA core loss or insufficient tissue. Hazard ratios (HRs) > 1 indicate an increased hazard of disease-specific death for the indicated category relative to the reference category, whereas HRs < 1 indicate a reduced hazard. Immune biomarkers were dichotomized into low- and high-density groups using the respective median value as the cut-off. Reference categories were age ≤ 60 years, female sex, non-smoking, no alcohol consumption, histological grade G1, pT1–2, pN0, UICC stage I–II, absence of lymphovascular invasion (LVI), absence of perineural invasion (PNI), and surgery without postoperative radiotherapy (PORT). HR, hazard ratio; CI, confidence interval.

**Table 7:** Univariate Cox proportional hazards regression analysis for disease-free survival.

Variable	HR	95%CI	*p* Value
Clinical and histological			
Age > 60 years	0.71	0.34–1.48	0.361
Male sex	3.01	0.41–22.17	0.278
Smoking	1.91	0.67–5.50	0.228
Alcohol consumption	1.71	0.81–3.61	0.163
Histological grade G2–G3	1.75	0.66–4.62	0.259
pT3–4	2.32	1.03–5.23	0.043
UICC stage III–IV	2.30	1.02–5.20	0.045
pN+	1.98	0.90–4.36	0.088
LVI positive	2.17	0.93–5.08	0.075
PNI positive	1.99	0.73–5.42	0.175
Postoperative radiotherapy (PORT)	1.54	0.73–3.25	0.262
Biomarkers			
CD68 intratumoral high	1.44	0.67–3.08	0.348
CD68 stromal high	1.18	0.56–2.52	0.663
CD163 intratumoral high	0.82	0.39–1.76	0.619
CD163 stromal high	1.66	0.79–3.51	0.185
FOXP3 intratumoral high	0.24	0.10–0.59	0.002
FOXP3 stromal high	0.48	0.21–1.06	0.070
CTLA4 intratumoral high	0.46	0.19–1.15	0.098
CTLA4 stromal high	0.26	0.11–0.61	0.002
FOXP3/CTLA4 intratumoral high	0.27	0.08–0.88	0.030
FOXP3/CTLA4 stromal high	0.56	0.25–1.25	0.158
PD1 intratumoral high	1.12	0.53–2.38	0.773
PD1 stromal high	0.81	0.38–1.73	0.592

Note: Univariate Cox proportional hazards regression analyses were performed for disease-free survival. A total of 29 recurrence events occurred in the complete LSCC cohort; the number of evaluable cases and events varied across individual biomarker analyses because of TMA core loss or insufficient tissue. Hazard ratios (HRs) > 1 indicate an increased hazard of recurrence for the indicated category relative to the reference category, whereas HRs < 1 indicate a reduced hazard. Immune biomarkers were dichotomized into low- and high-density groups using the respective median value as the cut-off. Reference categories were age ≤ 60 years, female sex, non-smoking, no alcohol consumption, histological grade G1, pT1–2, pN0, UICC stage I–II, absence of lymphovascular invasion (LVI), absence of perineural invasion (PNI), and surgery without postoperative radiotherapy (PORT). HR, hazard ratio; CI, confidence interval.

**Table 8:** Distribution of patients according to the exploratory Protective Immune Score (PIS).

Protective Immune Score (PIS)	Overall Cohort, *n* (%)	LSCC Cohort, *n* (%)
0	67 (38.1)	39 (34.2)
1	65 (36.9)	39 (34.2)
2	44 (25.0)	36 (31.6)

Note: The Protective Immune Score (PIS) was calculated as the sum of high intratumoral FOXP3-positive cell density and high stromal CTLA4-positive cell density (range, 0–2). One point was assigned for each biomarker. The score was calculated only for patients with evaluable results for both biomarkers. Data are presented as n (%). Abbreviation: LSCC, laryngeal squamous cell carcinoma.

**Table 9:** Multivariate Cox regression analysis for disease-specific survival.

Variable	Adjusted HR	95% CI	*p* Value
UICC stage III–IV	18.53	2.38–144.18	0.005
pN+	1.84	0.77–4.35	0.168
Protective Immune Score (PIS)	0.29	0.16–0.55	<0.001

Note: Multivariate Cox proportional hazards regression analysis was performed to evaluate the independent prognostic association of the Protective Immune Score (PIS) after adjustment for UICC stage and pathological nodal status. Reference categories were UICC stage I–II and pN0. HR, hazard ratio; CI, confidence interval.

**Table 10:** Multivariate Cox regression analysis for disease-free survival.

Variable	Adjusted HR	95% CI	*p* Value
UICC stage III–IV	2.57	0.99–6.69	0.052
pN+	1.70	0.70–4.13	0.238
Protective Immune Score (PIS)	0.29	0.17–0.52	<0.001

Note: Multivariate Cox proportional hazards regression analysis was performed to evaluate the independent prognostic association of the Protective Immune Score (PIS) after adjustment for UICC stage and pathological nodal status. Reference categories were UICC stage I–II and pN0. HR, hazard ratio; CI, confidence interval.

## Data Availability

The datasets analyzed during the current study are available from the corresponding author upon reasonable request.

## Supplementary Materials

The following supporting information can be downloaded at: https://www.mdpi.com/article/10.3390/cancers18193130/s1, Table S1: Clinical characteristics of patients with benign laryngeal polyps and laryngeal epithelial dysplasia; Table S2: Compartment-specific immune correlation analyses during laryngeal carcinogenesis. Complete compartment-specific correlation analyses are presented below. The main manuscript summarizes only the strongest biologically relevant correlations (Table 5 and Figure 2); Table S3: Complete Kaplan–Meier disease-specific survival analysis according to immune biomarker expression in the overall and advanced-stage LSCC cohorts; Table S4: Complete Kaplan–Meier disease-free survival analysis according to immune biomarker expression in the overall, advanced-stage, and early-stage LSCC cohorts.
