# Supplementary material for: Spatial Remodeling of the Immune Microenvironment During Laryngeal Carcinogenesis Reveals Prognostic Immune Patterns in Laryngeal Squamous Cell Carcinoma
Source: Cancers (Basel). 2026 Sep 27;18(19):3130. doi: 10.3390/cancers18193130 (PMC13664583; doi:10.3390/cancers18193130)
Supplement: Supplementary file 1 [file cancers-18-03130-s001.zip › Supplementary_Table_S1.pdf]

Supplementary Table S1. Clinical characteristics of patients with benign laryngeal polyps and laryngeal epithelial dysplasia

Variable	Benign laryngeal polyps (N = 45)	Laryngeal dysplasia (N = 55)
**Age, years, median (range)**	44 (26–78)	60.5 (24.7–85.1)
**Age, n (%)**		
≤60 years	39 (86.7)	27 (49.1)
>60 years	6 (13.3)	28 (50.9)
**Sex, n (%)**		
Male	39 (86.7)	40 (72.7)
Female	6 (13.3)	15 (27.3)
**Smoking, n (%)**		
Yes	NA	37 (67.3)
No	NA	18 (32.7)
**Alcohol consumption, n (%)**		
Yes	NA	5 (9.1)
No	NA	50 (90.9)
**Dysplasia grade, n (%)**	NA	
Low-grade	NA	29 (52.7)
High-grade	NA	26 (47.3)
**Progression to SCC, n (%)**	NA	
Non-progressors	NA	49 (89.1)
Progressors	NA	6 (10.9)

Note: Data are presented as n (%) unless otherwise indicated. NA, not available or not applicable; SCC, squamous cell carcinoma.
