# Supplementary material for: Spatial Remodeling of the Immune Microenvironment During Laryngeal Carcinogenesis Reveals Prognostic Immune Patterns in Laryngeal Squamous Cell Carcinoma
Source: Cancers (Basel). 2026 Sep 27;18(19):3130. doi: 10.3390/cancers18193130 (PMC13664583; doi:10.3390/cancers18193130)
Supplement: Supplementary file 1 [file cancers-18-03130-s001.zip › Supplementary_Table_S2.pdf]

Suplementary Table S2 Compartment-specific immune correlation analyses during laryngeal carcinogenesis. Complete compartment-specific correlation analyses are presented below. The main manuscript summarizes only the strongest biologically relevant correlations (Table 5 and Figure 2).

Group	Compartment	Marker pair	N	r_s	P value
**All samples**					
All samples	intraepithelial	PD1↔CD4	172	0.367	**<0.0001**
All samples	intraepithelial	PD1↔CD8	174	0.425	**<0.0001**
All samples	intraepithelial	PD1↔CD68	173	0.329	**<0.0001**
All samples	intraepithelial	PD1↔CD163	174	0.241	**0.0013**
All samples	intraepithelial	PD1↔FOXP3	168	0.124	0.109
All samples	intraepithelial	PD1↔CTLA4	172	0.306	**<0.0001**
All samples	intraepithelial	PD1↔FOXP3/CTLA4	167	0.075	0.333
All samples	intraepithelial	CTLA4↔CD4	170	0.204	**0.0076**
All samples	intraepithelial	CTLA4↔CD8	170	0.214	**0.005**
All samples	intraepithelial	CTLA4↔CD68	173	0.275	**0.0003**
All samples	intraepithelial	CTLA4↔CD163	173	0.222	**0.0034**
All samples	intraepithelial	CTLA4↔FOXP3	177	0.375	**<0.0001**
All samples	intraepithelial	CTLA4↔FOXP3/CTLA4	176	0.308	**<0.0001**
All samples	intraepithelial	FOXP3↔CD4	167	0.166	**0.032**
All samples	intraepithelial	FOXP3↔CD8	166	0.079	0.315
All samples	intraepithelial	FOXP3↔CD68	173	0.244	**0.0012**
All samples	intraepithelial	FOXP3↔CD163	171	0.256	**0.0007**
All samples	stromal	PD1↔CD4	167	0.561	**<0.0001**
All samples	stromal	PD1↔CD8	169	0.515	**<0.0001**
All samples	stromal	PD1↔CD68	169	0.313	**<0.0001**
All samples	stromal	PD1↔CD163	169	0.254	**0.0008**
All samples	stromal	PD1↔FOXP3	164	0.403	**<0.0001**
All samples	stromal	PD1↔CTLA4	167	0.292	**0.0001**
All samples	stromal	PD1↔FOXP3/CTLA4	164	0.354	**<0.0001**
All samples	stromal	CTLA4↔CD4	169	0.227	**0.0029**
All samples	stromal	CTLA4↔CD8	169	0.236	**0.0020**
All samples	stromal	CTLA4↔CD68	172	0.221	**0.0035**
All samples	stromal	CTLA4↔CD163	171	0.227	**0.0029**
All samples	stromal	CTLA4↔FOXP3	176	0.556	**<0.0001**
All samples	stromal	CTLA4↔FOXP3/CTLA4	176	0.571	**<0.0001**
All samples	stromal	FOXP3↔CD4	167	0.476	**<0.0001**

Group	Compartment	Marker pair	N	r_s	P value
All samples	stromal	FOXP3↔CD8	166	0.380	**<0.0001**
All samples	stromal	FOXP3↔CD68	173	0.330	**<0.0001**
All samples	stromal	FOXP3↔CD163	171	0.269	**0.0004**
**Dysplasia**					
All dysplasia	intraepithelial	PD1↔CD4	33	0.448	**0.009**
All dysplasia	intraepithelial	PD1↔CD8	32	0.361	**0.042**
All dysplasia	intraepithelial	PD1↔CD68	34	0.307	0.078
All dysplasia	intraepithelial	PD1↔CD163	33	0.295	0.096
All dysplasia	intraepithelial	PD1↔FOXP3	34	0.366	**0.033**
All dysplasia	intraepithelial	PD1↔CTLA4	34	0.433	**0.011**
All dysplasia	intraepithelial	PD1↔FOXP3/CTLA4	34	0.207	0.240
All dysplasia	intraepithelial	CTLA4↔CD4	35	0.130	0.456
All dysplasia	intraepithelial	CTLA4↔CD8	33	0.314	0.075
All dysplasia	intraepithelial	CTLA4↔CD68	35	0.306	0.074
All dysplasia	intraepithelial	CTLA4↔CD163	34	0.115	0.517
All dysplasia	intraepithelial	CTLA4↔FOXP3	36	0.249	0.143
All dysplasia	intraepithelial	CTLA4↔FOXP3/CTLA4	36	0.244	0.151
All dysplasia	intraepithelial	FOXP3↔CD4	35	0.032	0.857
All dysplasia	intraepithelial	FOXP3↔CD8	33	-0.031	0.865
All dysplasia	intraepithelial	FOXP3↔CD68	36	0.379	**0.023**
All dysplasia	intraepithelial	FOXP3↔CD163	35	0.521	**0.001**
All dysplasia	stromal	PD1↔CD4	31	0.654	**0.0001**
All dysplasia	stromal	PD1↔CD8	30	0.521	**0.003**
All dysplasia	stromal	PD1↔CD68	32	0.245	0.177
All dysplasia	stromal	PD1↔CD163	31	0.200	0.281
All dysplasia	stromal	PD1↔FOXP3	32	0.410	**0.019**
All dysplasia	stromal	PD1↔CTLA4	31	0.221	0.231
All dysplasia	stromal	PD1↔FOXP3/CTLA4	32	0.354	**0.047**
All dysplasia	stromal	CTLA4↔CD4	35	0.024	0.892
All dysplasia	stromal	CTLA4↔CD8	33	0.135	0.455
All dysplasia	stromal	CTLA4↔CD68	35	0.452	**0.006**
All dysplasia	stromal	CTLA4↔CD163	34	0.530	**0.001**
All dysplasia	stromal	CTLA4↔FOXP3	36	0.208	0.224
All dysplasia	stromal	CTLA4↔FOXP3/CTLA4	36	0.290	0.086

Group	Compartment	Marker pair	N	r_s	P value
All dysplasia	stromal	FOXP3↔CD4	35	0.319	0.062
All dysplasia	stromal	FOXP3↔CD8	33	0.247	0.166
All dysplasia	stromal	FOXP3↔CD68	36	0.279	0.099
All dysplasia	stromal	FOXP3↔CD163	35	0.070	0.690
Low-grade dysplasia	stromal	PD1↔CD4	16	0.707	**0.002**
Low-grade dysplasia	stromal	PD1↔CD8	14	0.668	**0.009**
Low-grade dysplasia	stromal	PD1↔CD68	17	0.342	0.179
Low-grade dysplasia	stromal	PD1↔CD163	16	0.117	0.665
Low-grade dysplasia	stromal	PD1↔FOXP3	16	-0.028	0.917
Low-grade dysplasia	stromal	PD1↔CTLA4	17	0.226	0.383
Low-grade dysplasia	stromal	PD1↔FOXP3/CTLA4	16	0.0009	0.997
Low-grade dysplasia	stromal	CTLA4↔CD4	20	-0.104	0.662
Low-grade dysplasia	stromal	CTLA4↔CD8	18	-0.088	0.727
Low-grade dysplasia	stromal	CTLA4↔CD68	21	0.573	**0.007**
Low-grade dysplasia	stromal	CTLA4↔CD163	20	0.653	**0.002**
Low-grade dysplasia	stromal	CTLA4↔FOXP3	21	-0.018	0.939
Low-grade dysplasia	stromal	CTLA4↔FOXP3/CTLA4	21	0.144	0.534
Low-grade dysplasia	stromal	FOXP3↔CD4	20	-0.215	0.364
Low-grade dysplasia	stromal	FOXP3↔CD8	18	-0.280	0.259
Low-grade dysplasia	stromal	FOXP3↔CD68	21	-0.191	0.408
Low-grade dysplasia	stromal	FOXP3↔CD163	20	-0.180	0.448
High-grade dysplasia	stromal	PD1↔CD4	15	0.424	0.115
High-grade dysplasia	stromal	PD1↔CD8	16	0.449	0.081
High-grade dysplasia	stromal	PD1↔CD68	15	-0.108	0.702
High-grade dysplasia	stromal	PD1↔CD163	15	-0.210	0.454
High-grade dysplasia	stromal	PD1↔FOXP3	16	0.479	0.061
High-grade dysplasia	stromal	PD1↔CTLA4	14	0.139	0.636
High-grade dysplasia	stromal	PD1↔FOXP3/CTLA4	16	0.221	0.410
High-grade dysplasia	stromal	CTLA4↔CD4	15	-0.075	0.791
High-grade dysplasia	stromal	CTLA4↔CD8	15	0.259	0.351
High-grade dysplasia	stromal	CTLA4↔CD68	14	0.105	0.721
High-grade dysplasia	stromal	CTLA4↔CD163	14	0.279	0.335
High-grade dysplasia	stromal	CTLA4↔FOXP3	15	0.269	0.332
High-grade dysplasia	stromal	CTLA4↔FOXP3/CTLA4	15	0.201	0.474

Group	Compartment	Marker pair	N	r_s	P value
High-grade dysplasia	stromal	FOXP3↔CD4	15	0.574	**0.025**
High-grade dysplasia	stromal	FOXP3↔CD8	15	0.570	**0.027**
High-grade dysplasia	stromal	FOXP3↔CD68	15	0.565	**0.028**
High-grade dysplasia	stromal	FOXP3↔CD163	15	0.159	0.572
**LSCC**					
All LSCC	intraepithelial	PD1↔CD4	105	0.124	0.208
All LSCC	intraepithelial	PD1↔CD8	108	0.334	**0.0004**
All LSCC	intraepithelial	PD1↔CD68	108	0.053	0.583
All LSCC	intraepithelial	PD1↔CD163	111	-0.023	0.814
All LSCC	intraepithelial	PD1↔FOXP3	106	0.054	0.581
All LSCC	intraepithelial	PD1↔CTLA4	110	0.225	**0.018**
All LSCC	intraepithelial	PD1↔FOXP3/CTLA4	106	0.081	0.409
All LSCC	intraepithelial	CTLA4↔CD4	108	0.225	**0.019**
All LSCC	intraepithelial	CTLA4↔CD8	110	0.253	**0.008**
All LSCC	intraepithelial	CTLA4↔CD68	113	0.172	0.069
All LSCC	intraepithelial	CTLA4↔CD163	114	0.173	0.065
All LSCC	intraepithelial	CTLA4↔FOXP3	114	0.421	**<0.0001**
All LSCC	intraepithelial	CTLA4↔FOXP3/CTLA4	114	0.368	**0.0001**
All LSCC	intraepithelial	FOXP3↔CD4	105	0.254	**0.009**
All LSCC	intraepithelial	FOXP3↔CD8	106	0.078	0.428
All LSCC	intraepithelial	FOXP3↔CD68	110	0.255	**0.007**
All LSCC	intraepithelial	FOXP3↔CD163	110	0.263	**0.006**
All LSCC	stromal	PD1↔CD4	104	0.387	**0.0001**
All LSCC	stromal	PD1↔CD8	107	0.384	**<0.0001**
All LSCC	stromal	PD1↔CD68	108	0.204	**0.034**
All LSCC	stromal	PD1↔CD163	110	0.125	0.194
All LSCC	stromal	PD1↔FOXP3	106	0.170	0.082
All LSCC	stromal	PD1↔CTLA4	110	0.158	0.099
All LSCC	stromal	PD1↔FOXP3/CTLA4	106	0.180	0.065
All LSCC	stromal	CTLA4↔CD4	108	0.112	0.249
All LSCC	stromal	CTLA4↔CD8	110	0.080	0.405
All LSCC	stromal	CTLA4↔CD68	113	-0.0008	0.994
All LSCC	stromal	CTLA4↔CD163	114	0.048	0.609
All LSCC	stromal	CTLA4↔FOXP3	114	0.471	**<0.0001**

Group	Compartment	Marker pair	N	r_s	P value
All LSCC	stromal	CTLA4↔FOXP3/CTLA4	114	0.483	**<0.0001**
All LSCC	stromal	FOXP3↔CD4	105	0.315	**0.001**
All LSCC	stromal	FOXP3↔CD8	106	0.176	0.071
All LSCC	stromal	FOXP3↔CD68	110	0.029	0.764
All LSCC	stromal	FOXP3↔CD163	110	-0.002	0.985
Early-stage LSCC	stromal	PD1↔CD4	41	0.232	0.145
Early-stage LSCC	stromal	PD1↔CD8	43	0.436	**0.004**
Early-stage LSCC	stromal	PD1↔CD68	44	0.358	**0.017**
Early-stage LSCC	stromal	PD1↔CD163	44	0.189	0.220
Early-stage LSCC	stromal	PD1↔FOXP3	45	0.102	0.507
Early-stage LSCC	stromal	PD1↔CTLA4	45	0.218	0.149
Early-stage LSCC	stromal	PD1↔FOXP3/CTLA4	45	0.089	0.557
Early-stage LSCC	stromal	CTLA4↔CD4	43	0.069	0.659
Early-stage LSCC	stromal	CTLA4↔CD8	45	0.096	0.531
Early-stage LSCC	stromal	CTLA4↔CD68	46	0.161	0.285
Early-stage LSCC	stromal	CTLA4↔CD163	46	0.164	0.277
Early-stage LSCC	stromal	CTLA4↔FOXP3	49	0.466	**0.0007**
Early-stage LSCC	stromal	CTLA4↔FOXP3/CTLA4	49	0.478	**0.0005**
Early-stage LSCC	stromal	FOXP3↔CD4	43	0.307	0.045
Early-stage LSCC	stromal	FOXP3↔CD8	45	0.219	0.148
Early-stage LSCC	stromal	FOXP3↔CD68	46	0.220	0.142
Early-stage LSCC	stromal	FOXP3↔CD163	46	0.158	0.293
Advanced-stage LSCC	stromal	PD1↔CD4	63	0.456	**0.0002**
Advanced-stage LSCC	stromal	PD1↔CD8	64	0.287	**0.022**
Advanced-stage LSCC	stromal	PD1↔CD68	64	0.115	0.367
Advanced-stage LSCC	stromal	PD1↔CD163	66	0.091	0.470
Advanced-stage LSCC	stromal	PD1↔FOXP3	61	0.137	0.291
Advanced-stage LSCC	stromal	PD1↔CTLA4	65	0.066	0.601
Advanced-stage LSCC	stromal	PD1↔FOXP3/CTLA4	61	0.196	0.131
Advanced-stage LSCC	stromal	CTLA4↔CD4	65	0.126	0.318
Advanced-stage LSCC	stromal	CTLA4↔CD8	65	0.042	0.739
Advanced-stage LSCC	stromal	CTLA4↔CD68	67	-0.069	0.577
Advanced-stage LSCC	stromal	CTLA4↔CD163	68	0.007	0.954
Advanced-stage LSCC	stromal	CTLA4↔FOXP3	65	0.486	**<0.0001**

Group	Compartment	Marker pair	N	r_s	P value
Advanced-stage LSCC	stromal	CTLA4↔FOXP3/CTLA4	65	0.465	**0.0001**
Advanced-stage LSCC	stromal	FOXP3↔CD4	62	0.293	**0.021**
Advanced-stage LSCC	stromal	FOXP3↔CD8	61	0.056	0.668
Advanced-stage LSCC	stromal	FOXP3↔CD68	64	-0.036	0.780
Advanced-stage LSCC	stromal	FOXP3↔CD163	64	-0.067	0.598

Note: Correlations were assessed using Spearman's rank correlation coefficient (*r*s). Marker pairs are shown using bidirectional notation (↔) to indicate positive associations without implying causality. Owing to tissue microarray core loss or insufficient tissue, the number of evaluable samples varied between analyses. Only stromal compartment analyses are presented for the low-grade and high-grade dysplasia and early- and advanced-stage LSCC subgroup analyses because these compartment-specific correlations were the most biologically relevant and informative.
