# Supplementary material for: Spatial Remodeling of the Immune Microenvironment During Laryngeal Carcinogenesis Reveals Prognostic Immune Patterns in Laryngeal Squamous Cell Carcinoma
Source: Cancers (Basel). 2026 Sep 27;18(19):3130. doi: 10.3390/cancers18193130 (PMC13664583; doi:10.3390/cancers18193130)
Supplement: Supplementary file 1 [file cancers-18-03130-s001.zip › Supplementary_Table_S3.pdf]

Supplementary Table S3. Complete Kaplan–Meier disease-specific survival analysis according to immune biomarker expression in the overall and advanced-stage LSCC cohorts.

Group	Variables (cut-off)	N	Cancer-specific deaths	Mean survival (months)	95% CI	Log-rank χ²	P value
Overall LSCC Group							
Overall LSCC Group	CD68 intratumoral (cut-off >2.9)					5.90	**0.015**
	Low	58	6	158.7	145.5-172.0		
	High	58	16	134.5	113.3-155.8		
Overall LSCC Group	CD68 stromal (cut-off >7.0)					0.39	0.532
	Low	63	10	151.7	135.7-167.7		
	High	53	12	145.9	126.1-165.6		
Overall LSCC Group	CD163 intratumoral (cut-off >2.5)					1.61	0.204
	Low	65	10	153.0	137.9-168.0		
	High	54	13	142.1	121.5-162.7		
Overall LSCC Group	CD163 stromal (cut-off >15.0)					1.36	0.243
	Low	83	13	151.5	137.3-165.7		
	High	36	10	139.9	115.7-164.1		
Overall LSCC Group	FOXP3 intratumoral (cut-off >0.17)					7.86	**0.005**
	Low	58	17	129.1	108.9 -149.2		
	High	56	5	168.6	154.1-183.1		
Overall LSCC Group	FOXP3 stromal (cut-off >3.5)					1.35	0.246
	Low	58	14	138.5	119.9-157.7		
	High	56	8	159.7	143.1-176.4		
Overall LSCC Group	CTLA4 intratumoral (cut-off >1.0)					0.92	0.339
	Low	77	16	142.9	127.0-158.7		
	High	41	6	158.2	137.9-178.6		
Overall LSCC Group	CTLA4 stromal (cut-off >4.0)					7.60	**0.006**
	Low	60	16	116.9	98.9-134.8		
	High	58	6	168.2	154.8-181.6		
Overall LSCC Group	PD1 intratumoral (cut-off >2.0)					0.08	0.776
	Low	61	11	152.8	135.2-170.4		
	High	54	12	134.2	116.8-151.5		
Overall LSCC Group	PD1 stromal (cut-off >4.42)					0.003	0.957
	Low	57	11	148.5	128.9-168.1		
	High	57	12	144.8	127.4-162.2		
Overall LSCC Group	FOXP3/CTLA4 intratumoral (cut-off >0)					1.58	0.209
	Low	79	18	145.5	129.2-161.8		
	High	35	4	123.8	108.5-139.0		
Overall LSCC Group	FOXP3/CTLA4 stromal (cut-off >1.0)					1.79	0.180
	Low	62	15	143.0	124.2-161.8		
	High	52	7	134.1	120.8-147.3		
Advanced-stage LSCC Group							
Advanced-stage LSCC Group	CD68 intratumoral (cut-off >2.9)					3.28	0.070
	Low	31	6	144.1	121.0-167.1		
	High	38	15	112.8	84.8-140.8		

Group	Variables (cut-off)	N	Cancer-specific deaths	Mean survival (months)	95% CI	Log-rank χ^2	P value
Advanced-stage LSCC Group	CD68 stromal (cut-off >7.0)					0.10	0.750
	Low	35	9	130.6	105.3-155.9		
	High	34	12	126.0	99.1-153.0		
Advanced-stage LSCC Group	CD163 intratumoral (cut-off >2.5)					0.07	0.791
	Low	34	10	121.0	96.2-145.8		
	High	37	12	127.5	100.8-154.2		
Advanced-stage LSCC Group	CD163 stromal (cut-off >15.0)					1.86	0.173
	Low	49	12	132.4	111.0-153.7		
	High	22	10	109.9	75.6-144.2		
Advanced-stage LSCC Group	FOXP3 intratumoral (cut-off >0.17)					9.13	**0.003**
	Low	32	16	94.4	66.3-122.4		
	High	33	5	156.5	133.0-179.9		
Advanced-stage LSCC Group	FOXP3 stromal (cut-off >3.5)					3.12	0.077
	Low	28	13	103.1	74.0-132.2		
	High	37	8	145.0	120.4-169.6		
Advanced-stage LSCC Group	CTLA4 intratumoral (cut-off >1.0)					0.33	0.566
	Low	47	15	122.6	100.4-144.7		
	High	22	6	136.8	104.0-169.6		
Advanced-stage LSCC Group	CTLA4 stromal (cut-off >4.0)					11.68	**0.0006**
	Low	32	16	84.8	58.3-111.3		
	High	37	6	158.2	137.9-178.4		
Advanced-stage LSCC Group	PD1 intratumoral (cut-off >2.0)					0.02	0.879
	Low	33	10	131.7	103.8-159.5		
	High	34	12	113.3	88.4-138.2		
Advanced-stage LSCC Group	PD1 stromal (cut-off >4.42)					0.03	0.855
	Low	30	10	129.9	93.2-154.7		
	High	37	12	118.5	95.0-141.9		
Advanced-stage LSCC Group	FOXP3/CTLA4 intratumoral (cut-off >0)					3.33	0.068
	Low	41	17	114.7	88.9-140.4		
	High	24	4	106.7	88.1-125.4		
Advanced-stage LSCC Group	FOXP3/CTLA4 stromal (cut-off >1.0)					3.15	0.076
	Low	35	15	112.8	84.8-140.7		
	High	30	6	114.6	95.6-133.5		

Note: Kaplan–Meier disease-specific survival (DSS) analyses according to immune biomarker expression. Patients were dichotomized into low- and high-expression groups using the predefined median cut-off for each biomarker. Survival distributions were compared using the log-rank test. Mean survival time and corresponding 95% confidence intervals (CI) are presented. The number of evaluable cases varied across biomarkers because of tissue microarray core loss or insufficient tissue.
