# Supplementary material for: Spatial Remodeling of the Immune Microenvironment During Laryngeal Carcinogenesis Reveals Prognostic Immune Patterns in Laryngeal Squamous Cell Carcinoma
Source: Cancers (Basel). 2026 Sep 27;18(19):3130. doi: 10.3390/cancers18193130 (PMC13664583; doi:10.3390/cancers18193130)
Supplement: Supplementary file 1 [file cancers-18-03130-s001.zip › Supplementary_Table_S4.pdf]

Supplementary Table S4. Complete Kaplan–Meier disease-free survival analysis according to immune biomarker expression in the overall, advanced-stage, and early-stage LSCC cohorts.

Group	Variables (cut-off)	N	Recurrences, N	Mean survival (months)	95% CI	Log-rank χ²	P value
Overall LSCC Group							
Overall LSCC Group	CD68 intratumoral (cut-off >2.9)					0.891	0.345
	Low	58	12	141.2	123.7- 158.7		
	High	58	15	137.4	116.5- 158.4		
Overall LSCC Group	CD68 stromal (cut-off >7.0)					0.190	0.663
	Low	63	13	141.8	123.4-160.1		
	High	63	14	139.4	118.5-160.2		
Overall LSCC Group	CD163 intratumoral (cut-off >2.5)					0.249	0.618
	Low	65	17	134.3	116.0-152.6		
	High	54	11	147.5	127.4-167.6		
Overall LSCC Group	CD163 stromal (cut-off >15.0)					1.799	0.179
	Low	83	16	145.1	129.7-160.4		
	High	36	12	127.9	101.3-154.5		
Overall LSCC Group	FOXP3 intratumoral (cut-off >0.17)					11.233	**0.0008**
	Low	58	21	115.1	93.4-136.9		
	High	56	6	166.5	151.8-181.2		
Overall LSCC Group	FOXP3 stromal (cut-off >3.5)					3.445	0.064
	Low	58	18	124.9	104.3-145.6		
	High	56	9	155.1	136.6-173.6		
Overall LSCC Group	CTLA4 intratumoral (cut-off >1.0)					2.879	0.089
	Low	71	21	130.0	112.3-147.8		
	High	44	6	159.6	140.2-179.0		
Overall LSCC Group	CTLA4 stromal (cut-off >4.0)					11.07	**0.0009**
	Low	60	20	103.7	84.0-123.4		
	High	58	7	165.6	151.5-179.7		
Overall LSCC Group	PD1 intratumoral (cut-off >2.0)					0.083	0.773
	Low	61	13	145.2	125.7-164.6		
	High	54	14	127.7	109.1-146.3		
Overall LSCC Group	PD1 stromal (cut-off >4.42)					0.289	0.591
	Low	57	14	139.3	118.4-160.2		
	High	57	13	139.9	121.0-158.7		
Overall LSCC Group	FOXP3/CTLA4 intratumoral (cut-off >0)					5.416	**0.020**
	Low	79	24	130.7	112.5-148.9		
	High	35	3	127.6	113.9-141.3		
Overall LSCC Group	FOXP3/CTLA4 stromal (cut-off >1.0)					2.055	0.152
	Low	62	18	132.6	112.1-153.0		
	High	52	9	128.1	113.2-143.1		
Advanced-stage LSCC Group							
Advanced-stage LSCC Group	CD68 intratumoral (cut-off >2.9)					0.614	0.433
	Low	31	8	133.0	107.3-158.6		
	High	38	12	125.3	97.4-153.2		

Group	Variables (cut-off)	N	Recurrences, N	Mean survival (months)	95% CI	Log-rank χ²	P value
Advanced-stage LSCC Group	CD68 stromal (cut-off >7.0)					1.296	0.255
	Low	35	7	139.9	115.9-163.7		
	High	34	13	120.4	92.5-148.3		
Advanced-stage LSCC Group	CD163 intratumoral (cut-off >2.5)					0.778	0.378
	Low	34	12	111.9	86.1-137.8		
	High	37	9	139.5	113.4-165.5		
Advanced-stage LSCC Group	CD163 stromal (cut-off >15.0)					1.201	0.273
	Low	49	12	133.4	112.5-154.2		
	High	22	9	112.7	76.7-148.7		
Advanced-stage LSCC Group	FOXP3 intratumoral (cut-off >0.17)					11.341	**0.0008**
	Low	32	16	90.6	61.6-119.7		
	High	33	4	163.1	142.3-184.0		
Advanced-stage LSCC Group	FOXP3 stromal (cut-off >3.5)					3.512	0.061
	Low	28	12	103.8	73.1-134.4		
	High	37	8	144.8	119.7-169.9		
Advanced-stage LSCC Group	CTLA4 intratumoral (cut-off >1.0)					0.718	0.397
	Low	47	15	120.7	97.8-143.6		
	High	22	5	144.6	112.9-176.2		
Advanced-stage LSCC Group	CTLA4 stromal (cut-off >4.0)					12.806	**0.0003**
	Low	32	15	81.7	54.2-109.2		
	High	37	5	162.7	143.8-181.6		
Advanced-stage LSCC Group	PD1 intratumoral (cut-off >2.0)					0.277	0.599
	Low	33	8	139.8	112.3-167.3		
	High	34	11	117.3	92.2-142.3		
Advanced-stage LSCC Group	PD1 stromal (cut-off >4.42)					0.177	0.674
	Low	30	9	130.4	100.4-160.3		
	High	37	10	124.2	100.5-147.9		
Advanced-stage LSCC Group	FOXP3/CTLA4 intratumoral (cut-off >0)					4.984	**0.026**
	Low	41	17	111.6	84.9-138.3		
	High	24	3	111.4	93.9-128.8		
Advanced-stage LSCC Group	FOXP3/CTLA4 stromal (cut-off >1.0)					2.864	0.091
	Low	35	14	113.9	85.0-142.7		
	High	30	6	114.5	95.2-133.8		
Early-stage LSCC Group							
Early-stage LSCC Group	CD68 intratumoral (cut-off >2.9)					0.02	0.891
	Low	27	4	134.3	115.1-153.4		
	High	20	3	154.4	121.7-181.2		
Early-stage LSCC Group	CD68 stromal (cut-off >7.0)					2.18	0.139
	Low	28	6	142.5	116.2-168.9		
	High	19	1	147.7	132.6-162.8		
Early-stage LSCC Group	CD163 intratumoral (cut-off >2.5)					0.06	0.807
	Low	31	5	152.5	131.0-174.0		
	High	17	2	159.3	132.2-186.3		

Group	Variables (cut-off)	N	Recurrences, N	Mean survival (months)	95% CI	Log-rank χ^2	P value
Early-stage LSCC Group	CD163 stromal (cut-off >15.0)					0.496	0.481
	Low	34	4	158.3	138.2-178.3		
	High	14	3	126.8	97.9-155.8		
Early-stage LSCC Group	FOXP3 intratumoral (cut-off >0.17)					1.36	0.244
	Low	26	5	145.1	117.8-172.3		
	High	23	2	144.2	129.0-159.3		
Early-stage LSCC Group	FOXP3 stromal (cut-off >3.5)					2.00	0.157
	Low	30	6	144.3	119.4-169.2		
	High	19	1	171.2	154.4-188.0		
Early-stage LSCC Group	CTLA4 intratumoral (cut-off >1.0)					2.25	0.133
	Low	30	6	142.9	117.2-168.7		
	High	19	1	171.7	155.8-187.6		
Early-stage LSCC Group	CTLA4 stromal (cut-off >4.0)					1.14	0.285
	Low	28	5	125.7	102.4-149.1		
	High	21	2	165.6	146.6-184.7		
Early-stage LSCC Group	PD1 intratumoral (cut-off >2.0)					0.22	0.637
	Low	28	5	147.5	121.9-173.1		
	High	20	3	106.9	90.6-123.4		
Early-stage LSCC Group	PD1 stromal (cut-off >4.42)					0.44	0.508
	Low	27	5	145.1	117.9-172.3		
	High	20	3	154.9	129.3-180.6		
Early-stage LSCC Group	FOXP3/CTLA4 intratumoral (cut-off >0)					2.09	0.149
	Low	38	7	147.7	126.2-169.1		
	High	11	0	140.3	140.3-140.3		
Early-stage LSCC Group	FOXP3/CTLA4 stromal (cut-off >1.0)					0.01	0.931
	Low	27	4	153.8	130.3-177.3		
	High	22	3	133.9	113.5-154.4		

Note: Kaplan–Meier disease-free survival (DFS) analyses according to immune biomarker expression. Patients were dichotomized into low- and high-expression groups using the predefined median cut-off for each biomarker. Disease-free survival distributions were compared using the log-rank test. Mean survival time without recurrence and corresponding 95% confidence intervals (CI) are presented. The number of evaluable cases varied across biomarkers because of tissue microarray core loss or insufficient tissue.
